# Microwave ablation compared with hepatic resection for the treatment of hepatocellular carcinoma and liver metastases: a systematic review and meta-analysis

**DOI:** 10.1186/s12957-019-1632-6

**Published:** 2019-06-10

**Authors:** Mrudula B. Glassberg, Sudip Ghosh, Jeffrey W. Clymer, George W. J. Wright, Nicole Ferko, Joseph F. Amaral

**Affiliations:** 1 Ethicon Inc., US-22, Somerville, NJ 08876 USA; 2 Ethicon Inc., 4545 Creek Rd, Cincinnati, OH 45242 USA; 3 Cornerstone Research Group Inc., 204-3228 South Service Road, Burlington, ON L7N 3H8 Canada

**Keywords:** Microwave ablation, Hepatic resection, Hepatocellular carcinoma, Meta-analysis

## Abstract

**Background:**

Hepatic resection (HR) is the gold standard liver cancer treatment, but few patients are eligible due to comorbidities or tumor location. Microwave ablation (MWA) is an important complementary liver cancer treatment to HR. This systematic review compared MWA with HR for liver cancer treatment.

**Methods:**

A systematic search of MEDLINE, EMBASE, and CENTRAL was conducted for randomized and observational studies published from 2006 onwards. The primary outcome was local tumor recurrence (LTR), and a random effects model was used for meta-analyses.

**Results:**

Of the 1845 studies identified, 1 randomized and 15 observational studies met the inclusion criteria. LTR was significantly increased with MWA versus HR (risk ratio (RR) = 2.49; *P* = 0.016). In secondary measures, HR provided significantly higher 3- and 5-year overall survival (RR = 0.94; *P* = 0.03 and RR = 0.88; *P* = 0.01, respectively) and 3-year disease-free survival (RR = 0.78; *P* = 0.009). MWA exhibited significantly shorter length of stay (weighted mean difference (WMD) = − 6.16 days; *P* < 0.001) and operative time (WMD = − 58.69 min; *P* < 0.001), less intraoperative blood loss (WMD = − 189.09 mL; *P* = 0.006), and fewer complications than HR (RR = 0.31; *P* < 0.001). When MWA was combined with HR and compared with either modality alone, complications and blood loss were significantly lower with the combination treatment; however, there were no differences in other outcomes. Subgroup and sensitivity analyses were generally aligned with the main results.

**Conclusions:**

MWA can be an effective and safe alternative to HR in patients/tumors that are not amenable to resection. More randomized and economic studies should be performed that compare the two treatments, especially to determine the target population that benefits most from MWA.

**Electronic supplementary material:**

The online version of this article (10.1186/s12957-019-1632-6) contains supplementary material, which is available to authorized users.

## Background

The incidence of liver cancer cases has been increasing, coupled with a 43% increase in mortality rate (10.3 per 100,000 in 2016 (USA)) [1, 2]. Liver cancer is also associated with one of the lowest 5-year survival rates among all types of cancer (19%) [1]. This means that identifying the safest and most effective treatments for liver cancer has never been more urgent.

Hepatic resection (HR) is the gold standard treatment for liver cancer for patients in whom surgery is not contraindicated and whose tumors are resectable [3]. The distinguishing factor between resectable and non-resectable tumors is whether a resection could be designed that would remove all residual disease with appropriate margins and leave the patient with sufficient liver remnant to support post-hepatectomy liver function [3, 4]. Most patients are not resectable (up to 80%) [3]; thus, HR is complemented by local ablative therapies such as radiofrequency ablation (RFA) and microwave ablation (MWA) for liver cancer treatment.

The use of MWA has increased dramatically over the last several years due to several advancements in the technology and the clinical application. MWA uses electromagnetic fields of either 915 or 2450 MHz to heat tissue to extreme temperatures resulting in the destruction of tumor cells surrounding the microwave antenna [5]. MWA is less invasive than HR [6, 7], but the margins achievable for tumors treated with HR are usually wider than those treated with MWA.

Recent meta-analyses of MWA with RFA for the treatment of liver cancer reported that MWA was at least as safe and effective as RFA [8, 9]. The Zhang et al. meta-analysis of nine hepatocellular carcinoma (HCC) studies showed that there were no differences between MWA and HR for recurrence, overall survival, and disease-free survival [10]. However, they showed that MWA was significantly favored over HR for operative time and blood loss, as well as complications [10]. Several studies have been published recently that were not included in the Zhang et al. meta-analysis, including those which compared HR and MWA in metastatic liver cancer [3, 4, 7, 11–17]. Furthermore, some outcomes were not analyzed by Zhang and colleagues including overall survival (OS) and disease-free survival (DFS) at multiple timepoints, intrahepatic de novo lesions (IDL), extrahepatic metastases (EHM), length of stay (LOS), and the proportion of patients with blood transfusions.

Due to the availability of new studies comparing MWA with HR for the treatment of liver cancer, as well as advancements in MWA technology and surgical techniques, a new meta-analysis is warranted to compare the two treatments. The objective of the current meta-analysis is to compare MWA and HR, alone or in combination with each other, across a comprehensive range of outcomes reported from both randomized and observational studies. Local tumor recurrence (LTR) is the primary outcome and secondary outcomes include 1-, 3-, and 5-year OS; IDL; EHM; operative time (min); intraoperative blood loss (mL); LOS (days); 1-, 3-, and 5-year disease-free survival (DFS); overall, major, and minor complications; and the proportion of patients with blood transfusions.

## Methods

### Search strategy

This systematic review and meta-analysis followed the Preferred Reporting Items for Systematic Reviews and Meta-Analyses (PRISMA) guidelines, and a PRISMA checklist can be found in Additional file 1 [18]. A systematic search of MEDLINE, EMBASE, and the Cochrane Central Register of Controlled Trials (CENTRAL) was conducted for relevant systematic reviews, randomized controlled trials (RCTs), and observational studies (prospective or retrospective cohort and case-control studies) using a search strategy developed by a medical information specialist that involved controlled vocabulary and keywords related to our research question (e.g., “Liver Neoplasms,” “Microwave,” “Ablation Techniques”) (Additional file 2). The search strategy was not limited by time or language; however, only English language articles published on or after January 1, 2006, were screened. The search strategy was also not initially limited to abstracts on resection, rather to MWA. The strategy was peer reviewed by another senior information specialist prior to execution using the PRESS checklist [19]. Three searches were performed: the first on October 29, 2017, for systematic reviews and RCTs, the second on November 24, 2017, for observational studies, and the third on March 16, 2018, as a combined update to the previous two searches. Reference lists of retrieved articles and relevant reviews were manually searched for additional studies.

### Study selection

Studies were selected for inclusion based on pre-defined PICOS criteria (i.e., population, intervention, comparator, outcomes, and study design). Studies were considered for inclusion in the meta-analysis if they were RCTs or observational studies comparing MWA with HR in adult patients (≥ 18 years) with confirmed HCC or liver cancer. These criteria were used to screen the titles and abstracts of publications to determine whether they were eligible for inclusion. Studies deemed eligible upon title and abstract screening were screened in full-text. Publications were reviewed in duplicate at each stage and discrepancies were resolved by consensus, or by adjudication by a third reviewer.

### Data extraction

Baseline characteristics and outcomes from the included studies were extracted using a standardized extraction form developed in Microsoft Excel. Key study characteristics were extracted. Some studies did not report percentages in text for OS and DFS but presented Kaplan–Meier (KM) curves that were digitized using DigitizeIt 2.2.2 (Braunschweig, Germany) and percentages extracted for 1-, 3-, and 5-year timepoints, where applicable. The numbers of surviving patients for OS and DFS were calculated by multiplying the percentage survival by the initial sample size for the treatment arm or the treatment group minus the excluded patients, where applicable. For outcomes where the standard deviation (SD) was not reported, SDs were imputed from the *P* value, which was used to calculate the *T*-score, standard error, and the SD [20]. For outcomes reported at different timepoints (other than OS and DFS at defined timepoints), the latest was extracted. Data were extracted by one reviewer and then examined for accuracy and completeness by a second reviewer.

### Study outcomes

The primary outcome was the rate of LTR, defined as local recurrence at or adjacent to the ablation site or the resection margin. Secondary outcomes were as follows: (1) 1-, 3-, and 5-year OS, (2) IDL, defined as the appearance of a new tumor at a new focus within the liver (sometimes defined as “intrahepatic recurrence” or “regional recurrence”), (3) EHM, defined as the appearance of new tumors outside the liver, (4) operative time (min), (5) intraoperative blood loss (mL), (6) length of stay (days), (7) 1-, 3-, and 5-year DFS, (8) complications, including any overall, major (e.g., Clavien–Dindo grade III or greater), or minor (e.g., Clavien–Dindo grade I or II) adverse events reported, and (9) proportion of patients with blood transfusions. (The proportion of patients with blood transfusions were only informed by studies for the HR versus MWA + HR comparison).

### Risk of bias assessment

The quality of studies included in the meta-analyses was assessed using the Cochrane Risk of Bias (RoB) tool [20] for RCTs and the Newcastle–Ottawa Quality Assessment Scale (NOS) [21] for observational studies.

For the NOS, studies were given a star if the patients enrolled were representative of the HCC population and classified as BCLC 0 or A [22]. For metastatic studies, there were no restrictions on primary origin. For comparability, HCC studies with treatment groups balanced on Child–Pugh classification, and metastatic studies with treatment groups balanced on tumor size and primary origin, received a star. If studies used matching, or regression analyses showed that Child–Pugh classification or tumor size and primary origin were not predictors of outcomes, they also received a star. Another star was given if additional effect modifiers were balanced or regression analyses showed these additional factors were not predictors of outcomes. Studies also received a star if the duration of follow-up was at least 6 months as well as when loss to follow-up was less than 20% [20]. A total of nine stars could be awarded to each study. The quality of included studies was assessed independently by two reviewers and reconciled by a third reviewer, if required.

### Data synthesis and statistical methods

The DerSimonian–Laird random effects model was used for the meta-analyses and forest plots were created. For continuous outcomes (i.e., operative time, intraoperative blood loss, and LOS), the weighted mean difference (WMD) and corresponding 95% confidence interval (CI) were calculated. For dichotomous outcomes (i.e., LTR, patients with blood transfusions, OS, DFS, IDL, EHM, and complications), the risk ratio (RR) and corresponding 95% CIs were calculated. All analyses were conducted for the combination of the RCT and observational studies.

*I*^2^ values were calculated to describe the percentage of variance attributable to heterogeneity between studies. The following ranges were used to interpret *I*^2^ values regarding the degree of heterogeneity present between the synthesized studies for each comparison: 0–40% represented minimal heterogeneity, 30–60% represented moderate heterogeneity, 50–90% represented substantial heterogeneity, and 75–90% represented considerable heterogeneity [20]. If *I*^2^ value was from 75 to 90%, but the CIs for the effect measures overlapped between studies, then the heterogeneity was classified as substantial. If the *I*^2^ was from 75 to 90% and the CIs for the effect measures did not overlap between studies, then the heterogeneity was classified as considerable. The following sub-group analyses were performed if there were at least two studies in each group: (1) tumor size (< 3 cm versus ≥ 3 cm), (2) type of liver tumor (HCC versus metastasis), and (3) microwave frequency (915 versus 2450 MHz). For the subgroup analysis on type of liver tumor, any study that included metastatic tumors was considered in the liver metastases subgroup. Similarly, Philips et al. [13] included some patients on whom MWA was performed with 915-MHz MWA ablation systems; thus, it was considered a 915-MHz study in the MWA frequency subgroup analysis. Sensitivity analyses were performed to assess alternative methods (i.e., fixed effects model), study quality (i.e., exclusion of lower-quality studies, defined as any RCT with high risk for any domain of the RoB tool or any observational study with ≤ 7 stars on the NOS), and exclusion of studies with imputed data. Publication bias was examined for LTR; 1-, 3-, and 5-year OS; and overall complications [23]. Data were analyzed using STATA (version 15.1, StataCorp LLC, Texas, USA).

## Results

**Fig. 1:**
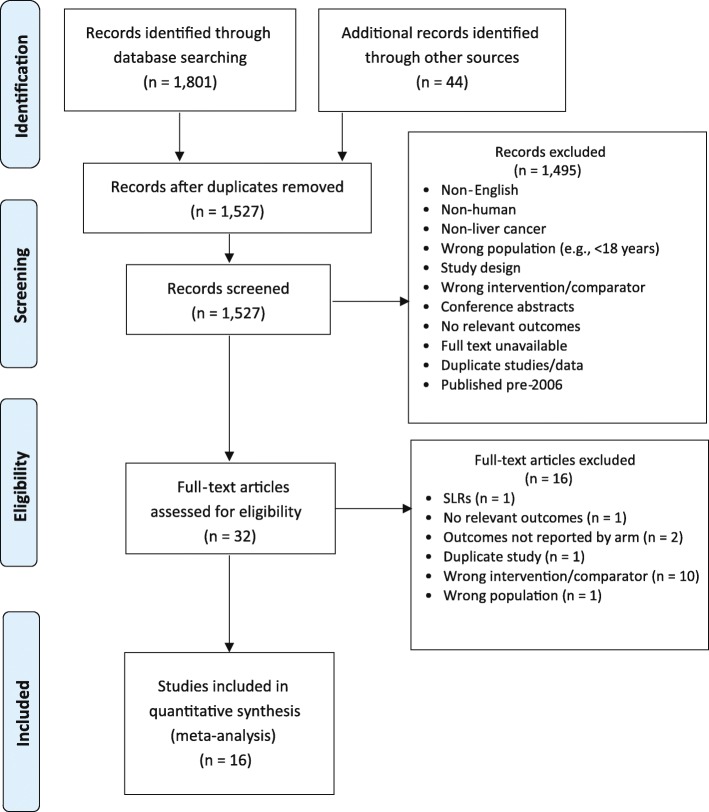
PRISMA flow diagram

A total of 1845 citations were identified from searches. After removing duplicates, 1527 unique records were screened. Of those, 1495 were excluded for various reasons (e.g., non-human, non-English, and not compared with surgery) (Fig. 1). Thirty-two articles were screened at the full-text stage. Of those, 16 were excluded because the studies reported the wrong intervention/comparator (*n* = 10), reported irrelevant outcomes (*n* = 1), did not report outcomes by treatment (*n* = 2), were systematic reviews (*n* = 1), were duplicates (*n* = 1), and involved the wrong population (*n* = 1). Sixteen studies that enrolled a total of 2522 patients were included in the meta-analysis [3, 4, 6, 7, 11–17, 24–28]. Study characteristics are presented in Table 1. The main analysis focused on the comparison of MWA with HR.

**Table 1:** Study and baseline characteristics for studies included in the meta-analysis

First author	Year	Study design	Region	Population	Arm 1	Arm 2	Arm 3	MWA approach	Patients (*N*)			Age (years)	Male (%)
									MWA	HR	MWA + HR		
Xu, J [23]	2015	RCT	China	HCC	MWA	HR	–	Percutaneous	45	45	–	58.1	73
Tanaka, K [4]	2006	Retrospective cohort	Japan	Metastases	–	HR	MWA + HR	–	–	37	16	60.3	62
Wang, ZL [24]	2008	Retrospective cohort	China	HCC	MWA	HR	–	Percutaneous	114	80	–	56.0	88
Imura, S [11]	2012	Prospective cohort	Japan	HCC	–	HR	MWA + HR	–	–	10	12	65.6	68
Stattner, S [3]	2013	Retrospective cohort	UK	Metastases	MWA	–	MWA + HR	Open	15	–	28	66.1	74
Takami, Y [25]	2013	Prospective cohort	Japan	HCC	MWA	HR	–	Thoracotomy, laparoscopy, or laparotomy	390	34	–	67.5	62
Shi, J [26]	2014	Retrospective cohort	China	HCC	MWA	HR	–	NR	117	107	–	55.6	80
Tan, K [12]	2014	Retrospective cohort	China	Metastases/ HCC*	–	HR	MWA + HR	–	–	62	66	50.3	84
Zhang, EL [6]	2016	Retrospective cohort	China	HCC	MWA	HR	–	Percutaneous	68	122	–	51.6	88
Li, W [27]	2017	Retrospective cohort	China	HCC	MWA	HR	–	Percutaneous, laparoscopic, and open	60	220	–	62.5	82
Philips, P [13]	2017	Retrospective cohort	USA	Metastases/ HCC*	MWA	HR	MWA + HR	Laparoscopic and open	108	84	84	61.0	55
Ryu, T [7]	2017	Retrospective cohort	Japan	Metastases	MWA	HR	MWA + HR	Open	13	14	7	66.0	71
Song, P [14]	2017	Retrospective cohort	China	Metastases	MWA	HR	–	Percutaneous†	28	34	–	60.0	53
Zhang, QB [15]	2017	Retrospective cohort	China	HCC	MWA	HR	–	Percutaneous	31	42	–	53.2	67
Chen, ZB [16]	2018	Retrospective cohort	China	HCC	–	HR	MWA + HR	–	–	191	112	51.6	73
Chong, CCN [17]	2018	Retrospective cohort	China	HCC	MWA	HR	–	Percutaneous, laparoscopic, and open	63	63	–	63.8	71

*Also included some patients with cholangiocarcinoma

†Assumed percutaneous because of the description of the temperature sensors

Abbreviations: *HCC* hepatocellular carcinoma, *HR* hepatic resection, *MWA* microwave ablation, *NR* not reported

The sample sizes of the included studies (one RCT and 15 observational studies) ranged from 22 to 424 patients, and the study follow-up durations ranged from 15 to 60 months. The average patient age across studies ranged from 50.3 to 67.5 years. Most studies focused on the comparison of MWA with HR alone; however, a few studies also compared MWA + HR with HR alone and/or MWA alone. Most of the studies originated from China (*n =* 10), and the other regions represented included Japan (*n =* 4), the USA (*n =* 1), and the UK (*n =* 1).

Some studies included patients that were nonresectable in the MWA treatment arm. Due to limited reporting and patient preference affecting which treatment was performed, calculating the number patients who were nonresectable was not possible. A table summarizing selection criteria can be found in Additional file 3. In brief, some but not all the included studies in our analysis mentioned selection criteria for each arm. When information was available, it was noted that HR was sometimes selected for patients with larger tumors and tumors near the surface. Microwave ablation was typically selected for patients with smaller and/or deeper tumors, more comorbidities, and a preference for a less-invasive procedure.

### Quality assessment

#### RCT

Risk of bias and study quality assessments for the single RCT included [24] are presented in the Appendix. The quality of the study by Xu and Zhao was acceptable as it had low or unclear risk of bias across all domains. Xu reported random number tables as the method of random sequence generation [24]. The methods used for allocation concealment were not stated and thus were classified as unclear risk of bias. Since the outcomes assessed by the study were objective, the risk of bias from blinding participants and personnel and outcome assessors were considered low. No patients were lost to follow-up and there were no missing data; hence, there was low risk of bias from incomplete outcome data. It was unclear whether bias from selective reporting was a factor and there was low risk of bias from any other sources.

#### Observational studies

The NOS assessments for observational studies are presented in Additional file 4, with scores that ranged from seven to nine stars. Studies varied in selection of the non-exposed cohort, comparability, suitable follow-up duration, and adequacy of follow-up. All studies included patients that were either truly or somewhat representative of the exposed cohort, used secure surgical records for the ascertainment of exposure, demonstrated that the outcome of interest was not present at the start of the study, and used links to surgical records to assess the outcomes. Two studies included some patients that were BCLC B rather than only BCLC 0 or A [15, 16]. Most studies drew the group receiving HR from the same source as the MWA or MWA + HR groups. However, some studies indicated that there were criteria for assigning patients with certain characteristics (e.g., age, tumor location, presence of comorbidities, tumor resectability, or patient/physician preference) to the different treatments [3, 6, 7, 13, 15, 17, 26, 28]. These studies were categorized as having the non-exposed cohort (HR group) drawn from a different source and were not given a star due to allocation bias. Most studies received two stars for comparability. However, two studies received only one star because they did not report Child–Pugh classification [11] or reported a difference in baseline Child–Pugh classification distribution and no regression analyses showing lack of effect on outcomes [28]. Every study was given a star for follow-up duration longer than 6 months, apart from one which was not long enough for LTR to occur [12]. Most studies had either complete follow-up of all patients or some loss to follow-up unlikely to bias results (i.e., < 20%). One study did not provide enough information to determine whether there was significant loss to follow-up and was not given a star [11].

### Analysis

#### Primary outcome (LTR)

**Fig. 2:**
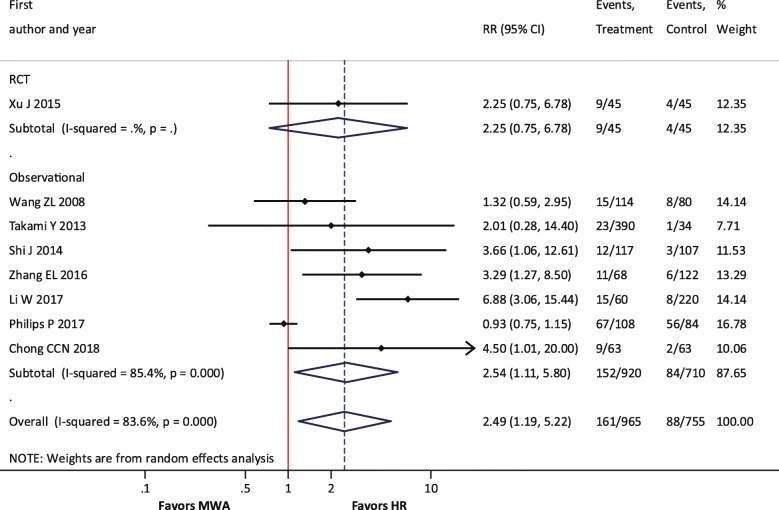
Forest plot of random effects meta-analysis results for LTR. Overall *P* = 0.016, stratified by RCTs (*P* = 0.15) versus observational studies (*P* = 0.027)

For LTR, one RCT and seven observational studies were included. The meta-analysis results demonstrated that the risk of LTR was significantly higher with MWA compared with HR (RR = 2.49; *P* = 0.016) (Table 2, Fig. 2). The single RCT of 90 patients did not show a significant difference in this outcome, although the RR of 2.25 was of a similar magnitude and direction. There were no differences in LTR between MWA + HR and HR (RR = 0.63; *P* = 0.263) or between MWA and MWA + HR (RR = 1.66; *P* = 0.549).

**Table 2:** Summary of analyses: MWA versus HR

Outcome	Number of studies included in meta-analysis	Summary effect* (95% CI); *P* value	Heterogeneity (*I*^2^ value) (%)
Primary outcome			
LTR	8	*2.49 (1.19, 5.22); P = 0.016*	84
Secondary outcomes			
OS (1-year)	10	1.01 (0.99, 1.03); *P* = 0.409	0
OS (3-year)	10	*0.94 (0.88, 0.99); P = 0.03*	0
OS (5-year)	9	*0.88 (0.80, 0.97); P = 0.01*	0
IDL	5	1.13 (0.80, 1.60); *P* = 0.474	75
EHM	2	1.10 (0.71, 1.72); *P* = 0.659	0
Operative time (min)	3	*− 58.69 (− 89.55, − 27.83); P < 0.001*	94
Intraoperative blood loss (mL)	3	*− 189.09 (− 324.54, − 53.64); P = 0.006*	93
Hospital length of stay (days)	6	*− 6.16 (− 8.25, − 4.07); P < 0.001*	84
DFS (1-year)	8	0.95 (0.90, 1.01); *P* = 0.085	39
DFS (3-year)	8	*0.78 (0.65, 0.94); P = 0.009*	59
DFS (5-year)	8	0.83 (0.58, 1.17); *P* = 0.284	71
Overall complications	9	*0.31 (0.19, 0.51); P < 0.001*	10
Major complications	4	*0.24 (0.10, 0.61); P = 0.002*	0
Minor complications	3	*0.45 (0.23, 0.90); P = 0.024*	66

*Risk ratio (RR) for MWA versus HR for all outcomes except operative time, intraoperative blood loss, and hospital length of stay, which are reported as the weighted mean difference (WMD). Italicized values indicate statistical significance. Point estimates and confidence intervals were calculated using a random effects model

Abbreviations: *CI* confidence interval, *DFS* disease-free survival, *EHM* extrahepatic metastasis, *HR* hepatic resection, *IDL* intrahepatic de novo lesions, *LTR* local tumor recurrence, *MWA* microwave ablation, *OS* overall survival, *RR* risk ratio

### Secondary outcomes

#### OS

**Fig. 3:**
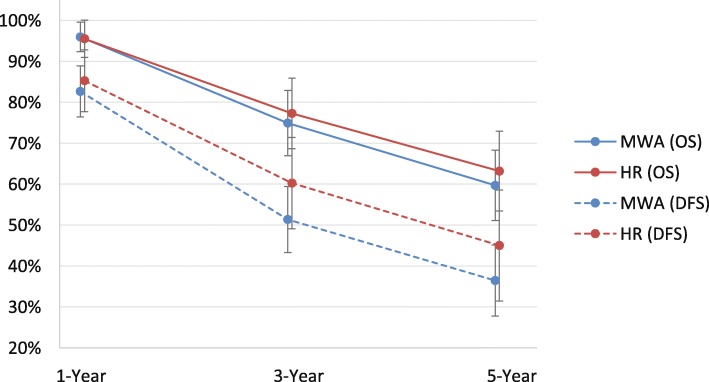
Weighted 1-, 3-, and 5-year overall survival and disease-free survival for MWA and HR. The error bars represent the 95% CIs for each estimate. Abbreviations: CI, confidence interval; DFS, disease-free survival; HR, hepatic resection; MWA, microwave ablation; OS, overall survival

**Fig. 4:**
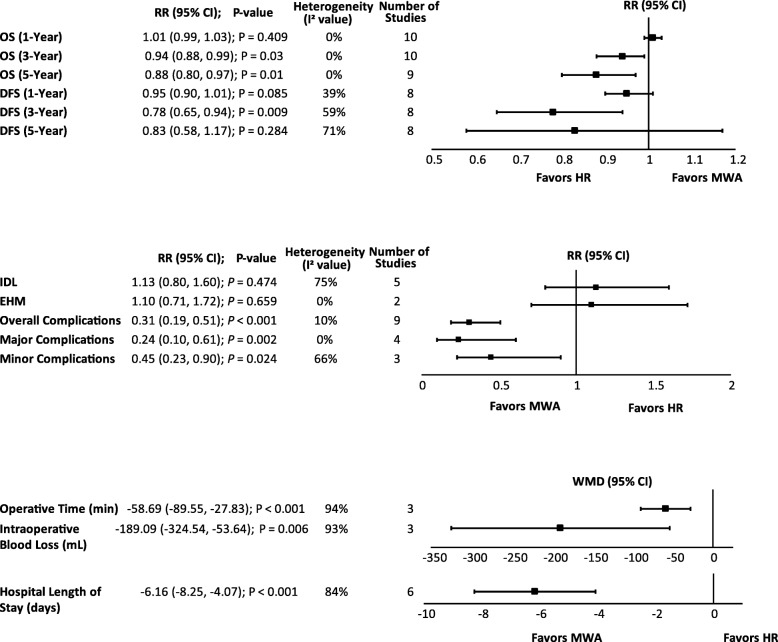
Summary of secondary analyses. Abbreviations: DFS, disease-free survival; EHM, extrahepatic metastases; HR, hepatic resection; IDL, intrahepatic de novo lesions; MWA, microwave ablation; OS, overall survival; RR, risk ratio; WMD, weighted mean difference

There was no significant difference between MWA and HR for 1-year OS (RR = 1.01; *P* = 0.409). However, 3- and 5-year OS were significantly higher with HR compared with MWA by 6% and 12%, respectively [3-year (RR = 0.94; *P* = 0.03) and 5-year OS (RR = 0.88; *P* = 0.01)] (Table 2, Fig. 4). The meta-analysis results were congruent with the weighted averages based on sample size of 1- (96.0% versus 95.5%), 3- (74.9% versus 77.3%), and 5-year (59.7% versus 63.2%) OS for MWA compared with HR (Fig. 3). For HR compared with MWA + HR, there were no significant differences in OS [1-year (RR = 0.99; *P* = 0.867), 3-year (RR = 0.84; *P* = 0.541), and 5-year OS (RR = 0.74; *P* = 0.651)] (Table 3). Similarly, for MWA + HR compared with MWA, there were no significant differences in OS [1-year (RR = 1.04; *P* = 0.495) and 3-year OS (RR = 1.24; *P* = 0.332)] (Table 4).

**Table 3:** Summary of analyses: MWA + HR versus HR

Outcome	Number of studies included in meta-analysis*	Summary effect† (95% CI); *P* value	Heterogeneity (*I*^2^ value) (%)
Primary outcome			
LTR	3	0.63 (0.28, 1.41); *P* = 0.263	69
Secondary outcomes			
OS (1-year)	4	0.99 (0.88, 1.11); *P* = 0.867	48
OS (3-year)	4	0.84 (0.49, 1.46); *P* = 0.541	82
OS (5-year)	2	0.74 (0.19, 2.79); *P* = 0.651	91
IDL	2	1.08 (0.61, 1.9); *P* = 0.793	51
EHM†	2	0.82 (0.49, 1.39); *P* = 0.464	27
Operative time (min)	4	13.93 (*−* 9.69, 37.54); *P* = 0.248	81
Intraoperative blood loss (mL)	4	*− 161.89 (− 291.49, − 32.30); P = 0.014*	92
Hospital length of stay (days)	2	0.08 (*−* 2.04, 2.19); *P* = 0.942	0
DFS (1-year)	3	0.95 (0.65, 1.40); *P* = 0.80	82
DFS (3-year)	3	1.01 (0.69, 1.48); *P* = 0.944	51
Overall complications	5	0.86 (0.63, 1.18); *P* = 0.353	0
Major complications	4	0.89 (0.57, 1.38); *P* = 0.588	12
Minor complications	3	0.83 (0.64, 1.08); *P* = 0.174	0
Blood transfusion	3	0.45 (0.14, 1.41); *P* = 0.171	85

*Three studies [11, 12, 16] included in this comparison used microwave radiation for pre-transection coagulation rather than tumor ablation

†Risk ratio (RR) for MWA + HR versus HR for all outcomes except operative time, intraoperative blood loss, and hospital length of stay, which are reported as the weighted mean difference (WMD). Italicized values indicate statistical significance. Point estimates and confidence intervals were calculated using a random effects model

Abbreviations: *CI* confidence interval, *DFS* disease-free survival, *EHM* extrahepatic metastasis, *IDL* intrahepatic de novo lesions, *HR* hepatic resection, *LTR* local tumor recurrence, *MWA* microwave ablation, *OS* overall survival, *RR* risk ratio

**Table 4:** Summary of analyses: MWA versus MWA + HR

Outcome	Number of studies included in meta-analysis	Summary effect* (95% CI); *P* value	Heterogeneity (*I*^2^ value) (%)
Primary outcome			
LTR	2	1.66 (0.32, 8.66); *P* = 0.103	62
Secondary outcomes			
OS (1-year)	3	1.04 (0.93, 1.17); *P* = 0.495	0
OS (3-year)	3	1.24 (0.81, 1.89); *P* = 0.322	11
Length of hospital stay (days)	2	− 1.98 (− 7.86, 3.90); *P* = 0.508	94
DFS (1-year)	2	0.78 (0.31, 1.94); *P* = 0.59	66
DFS (3-year)	2	0.59 (0.18, 1.91); *P* = 0.383	43
Overall complications	3	*0.39 (0.18, 0.85); P = 0.018*	20
Major complications	2	*0.24 (0.09, 0.60); P = 0.002*	0

*Risk ratio (RR) for MWA + HR versus HR for all outcomes except hospital length of stay, which is reported as the weighted mean difference (WMD). Italicized values indicate statistical significance. Point estimates and confidence intervals were calculated using a random effects model

Abbreviations: *CI* confidence interval, *DFS* disease-free survival, *HR* hepatic resection, *LTR* local tumor recurrence, *MWA* microwave ablation, *OS* overall survival, *RR* risk ratio

#### IDL and EHM

For IDL, there was no significant difference between MWA and HR (RR = 1.13; *P* = 0.474) (Table 2, Fig. 4). For EHM, there was also no significant difference between MWA and HR (RR = 1.10; *P* = 0.659) (Table 2, Fig. 4). The comparison of MWA + HR and HR exhibited similar patterns for both IDL (RR = 1.08; *P* = 0.793) and EHM (RR = 0.82; *P* = 0.464) (Table 3).

#### Operative time

There was a significant reduction of almost 1 h in the length of operative time with MWA compared with HR (WMD = − 58.69 min; *P* < 0.001) (Table 2, Fig. 4). In contrast, there was no significant difference in operative time between MWA + HR and HR (WMD = 13.93 min; *P* = 0.248) (Table 3).

#### Intraoperative blood loss

MWA was associated with a significant reduction in blood loss of 189.09 mL compared with HR (*P* = 0.006) (Table 2, Fig. 4). Intraoperative blood loss with MWA + HR was significantly reduced by 161.89 mL compared with HR (*P* = 0.014) (Table 3), driven by the three studies that used microwaves for pre-transection coagulation.

#### LOS

meta-analysis showed a significant LOS reduction of 6.16 days with MWA compared with HR (*P* < 0.001) (Table 2, Fig. 4). Only two studies reported LOS comparing MWA + HR and HR, and there was no significant difference between groups (WMD = 0.08 days; *P* = 0.942) (Table 3). Similarly, there was no significant difference in LOS between MWA and MWA + HR (WMD = − 1.98 days; *P* = 0.508) (Table 4).

#### DFS

The weighted averages of 1-, 3-, and 5-year DFS for MWA and HR are provided in Fig. 3. There were no significant differences between MWA and HR for 1-year (RR = 0.95; *P* = 0.085) and 5-year (RR = 0.83; *P* = 0.284) DFS. However, 3-year DFS was significantly lower for MWA compared with HR (RR = 0.78; *P* = 0.009) (Table 2, Fig. 4). For combination treatment comparisons, there were no significant differences between treatments for DFS ([MWA + HR versus HR: 1-year (RR = 0.95; *P* = 0.80) and 3-year (RR = 1.01; P = 0.944)] [MWA versus MWA +HR: 1-year (RR = 0.78; *P* = 0.59) and 3-year (RR = 0.59; *P* = 0.383)]) (Table 3, Table 4).

#### Overall, major, and minor complications

Overall complications were significantly reduced by 69% with MWA compared with HR in the main analysis (RR = 0.31; *P* < 0.001). A similar trend was observed with major complications (Clavien–Dindo class ≥ III) and minor complications (Clavien–Dindo class I or II), which were significantly reduced with MWA by 76% and 55%, respectively [major (RR = 0.24; *P* = 0.002) and minor complications (RR = 0.45; *P* = 0.024)] (Table 2, Fig. 4).

When comparing MWA alone to the combination of MWA + HR, MWA was shown to significantly reduce the risk of overall and major complications by 61% and 76%, respectively [overall (RR = 0.39; *P* = 0.018) and major complications (RR = 0.24; *P* = 0.002)] (Table 4). In contrast, when comparing HR alone to the combination of MWA + HR, there were no significant differences observed in overall, major, and minor complications [overall (RR = 0.86; *P* = 0.353), major (RR = 0.89; *P* = 0.588), and minor complications (RR = 0.83; *P* = 0.174)] (Table 3).

#### Blood transfusion

The proportion of patients who received blood transfusions was reported by three observational studies that compared MWA + HR with HR. Two of those studies also reported blood transfusion volume and an additional study reported duration of blood transfusion, which were excluded from the analyses. There was no significant difference in the proportion of patients who received blood transfusion between MWA + HR and HR groups (RR = 0.45; *P* = 0.171) (Table 3). Forest plots for all secondary analyses can be found online in Additional files 5, 6, 7, 8, 9, 10, 11, 12, 13, 14, 15, 16, 17, and 18.

### Subgroup analyses

#### Tumor size (< 3 cm versus ≥ 3 cm)

LTR was reported to be significantly higher with MWA than with HR for small tumors < 3 cm in diameter (RR = 2.93; *P* = 0.013), which aligned with the results of the main analysis. However, there was no significant difference in LTR between MWA and HR for larger tumors ≥ 3 cm in diameter (RR = 1.24; *P* = 0.624). For 1-year OS, there were no significant differences between groups regardless of tumor size. For overall complications, the significant reduction observed with MWA compared with HR in the main analysis was conserved for the ≥ 3-cm tumor group (RR = 0.30; *P* = 0.002); however, there were no significant differences between treatment arms in the < 3-cm tumor group (Table 5).

**Table 5:** Summary of subgroup analyses: MWA vs. HR

Subgroup	LTR [RR (95% CI); *P* value; studies (*N*); *I*^2^]	OS (1-year) [RR (95% CI); *P* value; studies (*N*); *I*^2^]	Complications [RR (95% CI); *P* value; studies (*N*); *I*^2^]
Tumor size			
< 3 cm	*2.93 (1.26, 6.81); P = 0.013; 4; 64%*	1.01 (0.99, 1.04); *P* = 0.41; 3; 0%	0.57 (0.06, 5.05); *P* = 0.615; 3; 67%
≥ 3 cm	1.24 (0.53, 2.88); *P* = 0.624; 2; 62%	0.96 (0.88, 1.04); *P* = 0.329; 3; 0%	*0.30 (0.14, 0.64); P = 0.002; 2; 0%*
Type of tumor			
HCC	*3.04 (1.84, 5.02); P < 0.001; 7; 32%*	1.01 (0.99, 1.03); *P* = 0.311; 7; 0%	*0.36 (0.19, 0.66); P = 0.001; 6; 21%*
Liver metastases	Too few studies (< 2) to inform	0.96 (0.88, 1.06); *P* = 0.451; 3; 0%	*0.22 (0.09, 0.56); P = 0.001; 2; 0%*
MWA frequency			
2450 MHz	*2.35 (1.49, 3.70); P < 0.001; 6; 0%*	1.00 (0.98, 1.03); *P* = 0.766; 8; 0%	*0.35 (0.18, 0.68); P = 0.002; 6; 31%*
915 MHz	2.44 (0.31, 19.10); *P* = 0.396; 2; 96%	0.98 (0.84, 1.14); *P* = 0.77; 2; 83%	*0.24 (0.10, 0.61); P = 0.002; 2; 0%*

Italicized values indicate statistical significance. Point estimates and confidence intervals were calculated using a random effects model

Abbreviations: *CI* confidence interval, *HCC* hepatocellular carcinoma, *LTR* local tumor recurrence, *MWA* microwave ablation, *OS* overall survival, *RR* risk ratio

#### Type of tumor (HCC versus metastases)

MWA had significantly higher risk of LTR compared with HR for HCC tumors (RR = 3.04; *P* < 0.001). For 1-year OS, there were no significant differences between MWA and HR regardless of tumor type. There were significantly fewer overall complications with MWA compared with HR regardless of tumor type [HCC (RR = 0.36; *P* = 0.001) versus metastases (RR = 0.22; *P* = 0.001)] (Table 5).

#### MWA frequency (915 versus 2450 MHz)

LTR was higher with MWA than HR for tumors treated with 2450 MHz MWA (RR = 2.35; *P* < 0.001), but there was no significant difference between MWA and HR with 915-MHz MWA (RR = 2.44; *P* = 0.396). There were also no significant differences in 1-year OS regardless of the MWA frequency used. There were significantly fewer overall complications with MWA compared with HR regardless of the MWA frequency used [2450 MHz (RR = 0.35; *P* = 0.002) versus 915 MHz (RR = 0.24; *P* = 0.002)] (Table 5).

### Sensitivity analyses

The results from the sensitivity analyses on alternative methods (i.e., fixed effects model), exclusion of poor-quality studies (i.e., those that received ≤ 7 stars on NOS assessment, the RCT by Xu and Zhao was not excluded for quality), and exclusion of studies where data were imputed (i.e., studies where missing SDs for continuous outcomes were calculated [13, 28]) were similar in magnitude and direction to the main analyses with only a few exceptions. For example, using a fixed effects model increased the number of DFS timepoints with which HR was significantly higher than MWA. Furthermore, when lower-quality studies were excluded, the risk of minor complications was no longer significantly different between treatment groups. Detailed results are presented in Table 6.

**Table 6:** Summary of sensitivity analyses

	Main analysis [RR (95% CI); *P* value; studies (*N*); *I*^2^]	Fixed effects [RR (95% CI); *P* value; studies (*N*); *I*^2^]	Exclusion of poor quality studies [RR (95% CI); *P* value; studies (*N*); *I*^2^]	Exclusion of studies with imputed data [RR (95% CI); *P* value; studies (*N*); *I*^2^]
LTR	*2.49 (1.19, 5.22); P = 0.016; 8; 84%*	*1.56 (1.28, 1.90); P < 0.001; 8; 84%*	*2.01 (1.06, 3.84); P = 0.033; 7; 72%*	NA
OS (1-year)	1.01 (0.99, 1.03); *P* = 0.409; 10; 0%	1.00 (0.97, 1.03); *P* = 0.977; 10; 0%	1.00 (0.97, 1.03); *P* = 0.992; 9; 0%	NA
OS (3-year)	*0.94 (0.88, 0.99); P = 0.03; 10; 0%*	*0.92 (0.86, 0.99); P = 0.016; 10; 0%*	0.94 (0.88, 1.00); *P* = 0.061; 9; 0%	NA
OS (5-year)	*0.88 (0.80, 0.97); P = 0.01; 9; 0%*	*0.87 (0.79, 0.96); P = 0.008; 9; 0%*	*0.88 (0.79, 0.98); P = 0.025; 8; 0%*	NA
IDL	1.13 (0.80, 1.60); *P* = 0.474; 5; 75%	1.12 (0.95, 1.33); *P* = 0.17; 5; 75%	1.14 (0.74, 1.76); *P* = 0.544; 4; 81%	NA
EHM	1.10 (0.71, 1.72); *P* = 0.659; 2; 0%	1.09 (0.70, 1.69); *P* = 0.71; 2; 0%	Too few studies (< 2) to inform	NA
Operative time (min)*	*− 58.69 (− 89.55, − 27.83); P < 0.001; 3; 94%*	*− 67.99 (− 74.36, − 61.61); P < 0.001; 3; 94%*	*− 44.61 (− 63.74, − 25.49); P < 0.001; 2; 49%*	*− 58.89 (− 100.15, − 17.63); P = 0.005; 2; 97%*
IBL (mL)*	*− 189.09 (− 324.54, − 53.64); P = 0.006; 3; 93%*	*− 119.88 (− 146.57, − 91.19); P < 0.001; 3; 93%*	*− 189.09 (− 324.54, − 53.64); P = 0.006; 3; 93%*	*−* 229.28 (*−* 512.69, 54.12); *P* = 0.113; 2; 97%
LOS (days)*	*− 6.16 (− 8.25, − 4.07); P < 0.001; 6; 84%*	*− 7.13 (− 7.78, − 6.48); P < 0.001; 6; 84%*	*− 6.14 (− 8.60, − 3.68); P < 0.001; 5; 87%*	*− 6.5 (− 9.52, − 3.48); P < 0.001; 4; 88%*
DFS (1-year)	0.95 (0.90, 1.01); *P* = 0.085; 8; 39%	*0.94 (0.89, 0.99); P = 0.016; 8; 39%*	0.95 (0.90, 1.01); *P* = 0.085; 8; 39%	NA
DFS (3-year)	*0.78 (0.65, 0.94); P = 0.009; 8; 59%*	*0.76 (0.68, 0.85); P < 0.001; 8; 59%*	*0.78 (0.65, 0.94); P = 0.009; 8; 59%*	NA
DFS (5-year)	0.83 (0.58, 1.17); *P* = 0.284; 8; 71%	*0.79 (0.66, 0.95); P = 0.011; 8; 71%*	0.83 (0.58, 1.17); *P* = 0.284; 8; 71%	NA
Overall complications	*0.31 (0.19, 0.51); P < 0.001; 9; 10%*	*0.33 (0.21, 0.50); P < 0.001; 9; 10%*	*0.32 (0.19,0.55); P < 0.001; 8; 19%*	NA
Major complications	*0.24 (0.10, 0.61); P = 0.002; 4; 0%*	*0.23 (0.09, 0.59); P = 0.002; 4; 0%*	Too few studies (< 2) to inform	NA
Minor complications	*0.45 (0.23, 0.90); P = 0.024; 3; 66%*	*0.43 (0.32, 0.59); P < 0.001; 3; 66%*	1.32 (0.04, 39.86); *P* = 0.872; 2; 83%	NA

*The effect measures for operative time, intraoperative blood loss, and LOS are the weighted mean differences.

Italicized values indicate statistical significance. Point estimates and confidence intervals were calculated using a random effects model apart from the sensitivity using a fixed effects model

Abbreviations: *CI* confidence interval, *DFS* disease-free survival, *EHM* extrahepatic metastases, *HCC* hepatocellular carcinoma, *IBL* intraoperative blood loss, *IDL* intrahepatic de novo lesions, *LOS*, length of stay, *LTR* local tumor recurrence, *MWA* microwave ablation, *OS* overall survival, *RR* risk ratio

### Publication bias

**Fig. 5:**
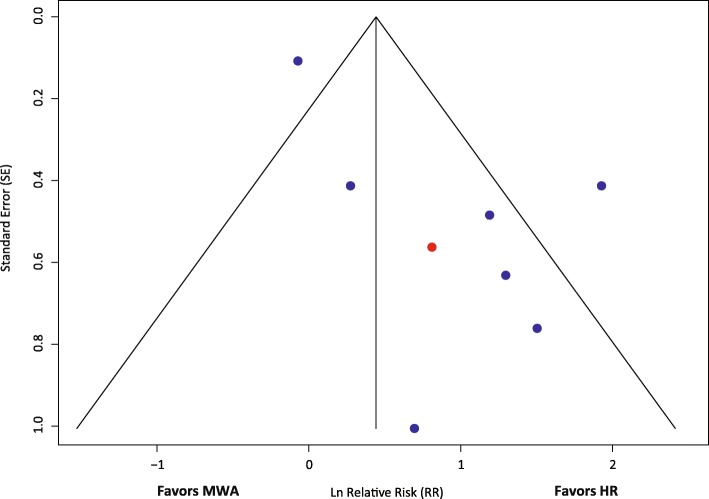
Funnel plot assessing publication bias for LTR in eight studies. The red dot indicates the RCT. Abbreviations: HR, hepatic resection; MWA, microwave ablation

The funnel plots demonstrated a slight risk of publication bias for LTR in favor of HR, but a low risk of bias for the other outcomes that were assessed. The funnel plot for LTR is presented in Fig. 5, and those for other outcomes are presented online in Additional file 19.

## Discussion

The results of this meta-analysis of 16 studies demonstrate that MWA can be an effective and safe alternative to HR and that each modality has its advantages in liver cancer treatment. Overall, HR was significantly favored over MWA for the primary outcome of LTR. For OS, DFS, IDL, and EHM outcomes, many results showed no significant differences between groups; however, HR was significantly favored over MWA for 3- and 5-year OS and 3-year DFS. For complication and resource-related outcomes, MWA was significantly favored over HR for operative time, intraoperative blood loss, hospital length of stay, and overall, major, and minor complications. These results remained similar with several alternative scenarios.

Identifying which patients would benefit most from each type of treatment is an important clinical challenge. MWA is less invasive than HR and can be preferentially performed on patients for whom surgery is contraindicated because of age or comorbidities [6, 7]. Poor liver function and/or cirrhosis is an important comorbidity that was sometimes considered in these studies as a selection criteria for MWA over HR. [6, 17] Portal hypertension is another comorbidity that makes patients poor candidates for HR, especially because blood loss can cause additional complications [6]. Non-resectable patients may also be classified as such for reasons unrelated to disease severity (e.g., a deeply located tumor). MWA is easier to implement for lesions located in the center of the liver than HR [7, 24] and requires less sacrifice of normal liver tissue [6]. Another advantage of MWA is that it can treat multiple nodules at the same time [26] less invasively than HR, which may require a two-staged approach, depending on tumor location. Hence, few patients with multiple HCC are indicated for HR. [26] Often it is the distribution of multiple tumors, rather than tumor size alone, that makes patients non-resectable [13]. However, larger liver tumors (including metastases) may be better suited for HR [7, 25], as treating them with MWA would require multiple overlapping ablations, increasing treatment difficulty and thereby yielding less-precise ablation margins than resection [27]. Resection is also the preferred treatment for tumors on the surface or edges of the liver [7]. For patients with colorectal liver metastases, HR may present a disease cure [3]. However, there are cases where complete HR is not possible in a single procedure. Aggressive use of MWA combined with HR may increase the proportion of patients eligible for resection [4]. MWA and HR are complementary techniques, and each treatment should be selected after considering its strengths and limitations, as well as several patient and tumor factors [7].

Given the selection criteria sometimes present in these studies, MWA-treated patients may have had worse clinical presentations than those treated with HR. As mentioned, some studies included patients in the MWA arm whose tumors were not reachable or not amenable to HR [3, 6, 13, 17, 28], for instance those located deep within the liver [6] or near the main hepatic outflow [3]. Several studies included patients in the MWA treatment arm whose tumors were non-resectable because of comorbidities or poor liver function [4, 6, 7, 13, 26, 28]. Wang et al. showed that age > 60 years was an independent prognostic risk factor that unfavorably affected disease-free survival [25]. Another study showed that the severity of liver cirrhosis was an independent risk factor for OS [6], both of which could have affected the study results. Considering these factors, MWA may have been disadvantaged by the disproportionate assignment of patients with comorbidities, poor liver function, and older age to it rather than HR. However, despite potential imbalances, the study populations were reasonably comparable and approximately half of the studies reported no differences in baseline characteristics between treatment arms [11, 12, 15–17, 24, 25, 27]. Also, MWA was not always used on patients ineligible for surgery. Additional study may help confirm the effects of selection bias.

A recent meta-analysis compared MWA with HR for the treatment of HCC and included nine observational studies from China or Japan [10]. These results reported by Zhang et al. showed that there were no significant differences between MWA and HR for recurrence, OS, and DFS [10]. Unlike Zhang, our current meta-analysis found that HR showed significantly lower local tumor recurrence than MWA. These discrepant findings may be because more studies were included in our meta-analysis, and there were some methodological study differences. The local recurrence definition used in our study was more specific than that of Zhang et al., who included all types of recurrence, local and distant [10]. Notably, recurrence or new tumors reported by Wang and colleagues favored MWA, but new tumors at the ablation site/resection line (local recurrence) favored HR. [25] Zhang et al. also used hazard ratios for the meta-analysis of OS and DFS which were not used here because the survival curves violated the proportional hazards assumption. Since the curves crossed, often multiple times, comparisons using hazard ratios were deemed to be inappropriate. As such, RR at 1-, 3-, and 5-year timepoints were used in our meta-analysis instead. Finally, results from Zhang regarding operative time, intraoperative blood loss, and complications aligned with our results [10].

The primary outcome of this meta-analysis showed that HR significantly reduced the risk of LTR compared with MWA. This result was expected since HR enables wider margins (> 1 cm) than MWA. One of the included studies showed that margins less than 1 cm was an independent risk factor for early recurrence [27]. Wider margins with HR may enable removal of microscopic tumor that may extend beyond 1 cm from the macroscopic border of even a small tumor [6, 27], which would not necessarily be ablated by MWA with standard 1-cm margins [6]. Xu and Zhao found that there were no differences between MWA and HR in overall recurrence, but there was significantly higher local recurrence with MWA than HR, the possible cause of which they attributed to larger tumor sizes in the MWA group [24]. Wang et al., reported that overall recurrence of new tumors was higher in the HR group (76.3%) than in the MWA group (70.2%), but local recurrence was higher in the MWA group compared with that in the HR group [25]. Thus, although HR exhibited significantly lower LTR rates than MWA, likely because of wider margins, there may be other factors that affect tumor recurrence beyond the original site.

Six of the included studies reported re-treatment of recurrent tumors during follow-up [6, 7, 15, 17, 26, 27]. When overall survival outcomes were similar between HR and MWA, this may be partially related to retreatment of recurrence [27, 28]. Since this analysis showed that LTR is more likely with MWA than HR, the efficacy of MWA for extending overall survival may be enhanced by retreatment compared with HR. [28] One advantage of initial treatment with MWA is that retreatment options are broader compared with HR. Parenchyma-sparing techniques like MWA reduce liver damage and preserve functional liver volume, maximizing the opportunity for subsequent liver-directed treatments [3, 24]. Patients with LTR who initially received HR may not be able to receive subsequent HR due to their residual liver volume being too small. Of note, local ablation techniques, including MWA and RFA, were the most common modalities used by the studies that reported retreatment method counts, including for patients who were initially treated with HR. [6, 7, 15, 17, 27] It was not possible to control for the potential effects of retreatment in these analyses and it is unknown whether the results for MWA would have been better if all studies had adequately retreated local recurrence.

Most of the studies included in this meta-analysis used microwave generators and antennas for tumor ablation [3, 4, 6, 7, 13–15, 17, 24–28]. However, three studies included in the MWA + HR versus HR comparison used microwave antennas for the purpose of pre-transection coagulation [11, 12, 16]. Two of them used a pre-transection coagulation technique involving sequentially inserting a microwave antenna along the transection plane and applying microwave radiation to coagulate blood vessels, prior to removal of the affected section [12, 16]. The aim of these procedures was to prevent blood loss and dissemination of tumors through the portal venous system [11, 16]. Intraoperative blood loss and post-operative liver failure are the two main risks of HR, especially in cirrhotic patients [11]. In one study, patients who received MW + HR for pre-transection coagulation did not require blood transfusions [11]. Additionally, the meta-analysis showed a statistically significant reduction in intraoperative blood loss, especially for studies that used MW for pre-transection coagulation [11, 12, 16]. However, there was no significant difference in the proportion of patients who received blood transfusions.

An important result of this meta-analysis was the consistently significant reductions in complications with MWA compared with HR. Reducing complications is an important performance goal as it improves patient care and reduces treatment costs. The definition of overall complications within the studies of the meta-analysis often included one or more of the following events: pleural effusion [6, 14–16, 24, 25], perihepatic effusion [14], inferior diaphragmatic effusion [15], ascites [6, 11, 15, 16], postoperative blood loss [6, 16, 24], bile leakage [6, 11, 13, 16, 24], portal vein thrombosis [15], hyperbilirubinemia [4], cardiopulmonary insufficiency [15], liver abscess [7, 14, 16], cholangitis [14], wound infection with or without dehiscence [4, 6, 13, 16, 25], pneumonia [6, 13], hepatic encephalopathy [6], biliary fistula [4], and intestinal obstruction or ileus [4, 13]. All of the studies that reported complications showed reduced rates with MWA compared with HR (and MWA compared with MWA + HR), apart from Wang and colleagues who reported six cases of minor pleural effusion in the MWA arm and no minor or severe complications in the HR arm [25]. It is unclear whether this is because of selective reporting. MWA also significantly reduced intraoperative blood loss compared with HR, which is an important consideration for reducing morbidity in patients with comorbidities, such as cirrhosis or portal hypertension. In summary, since MWA significantly reduced overall, major, and minor complications compared with HR, it can be considered as a safer alternative in appropriately indicated patients.

The favorable results for MWA involving significantly reduced LOS and operative time can be very important from a costing perspective. A recent US study of hepatic resection quoted an operating room cost of $11,958 for hepatic resection, with a mean operating time of 256 min, approximating to a per minute cost of $50 [29]. Thus, saving close to 60 min of operative time may save close to $3,000 per procedure with MWA compared with HR. This may underestimate potential savings as it does not account for fewer complications or shorter LOS with MWA. Reductions in LOS with MWA would free beds for other patients and improve hospital throughput. Song and colleagues reported that the costs associated with HR were significantly reduced by almost one half with MWA [14]. None of the other included studies performed economic analyses. Cost-effectiveness studies examining MWA versus HR have not been published to our knowledge, but an upcoming RCT on thermal ablation versus HR for liver metastases included cost-effectiveness analyses in its protocol [30]. A study examining the cost-effectiveness of RFA versus HR found that RFA was significantly more cost-effective for all tumors meeting Milan criteria than HR. [31] Given these results, economic studies of MWA are warranted. Future cost-effectiveness analyses should consider a multitude of parameters (e.g., supplies, operating time, length of stay, complications, follow-up tests (e.g., MRI), and visits) to comprehensively assess the total resource implications involving less-invasive therapies compared with resection for liver tumors. Such studies should also consider an appropriate time horizon based on the span of time that resource utilization differences between treatments is anticipated to occur.

**Fig. 6:**
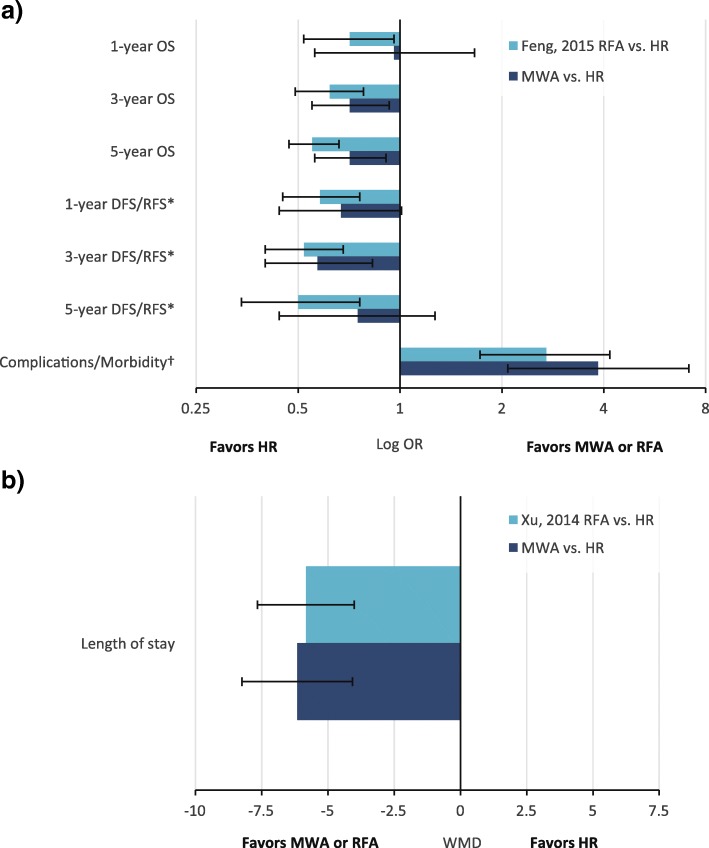
Results comparison for RFA vs. HR meta-analyses [31, 32] with current study for **a** categorical and **b** continuous outcomes. *Feng, 2015 reported recurrence-free survival, where DFS was reported by the current study. †The ORs for complications have been inverted so that the favored treatment labels agree with those for the survival outcomes. ORs were calculated for the current study (MWA vs. HR) for comparability with outcome measures from Feng, 2015. Abbreviations: DFS, disease-free survival; HR, hepatic resection; MWA, microwave ablation; OR, odds ratio; OS, overall survival; RFA, radiofrequency ablation; RFS; recurrence-free survival; WMD, weighted mean difference

Recent meta-analyses have examined the comparison of RFA versus HR for the treatment of liver cancer [32, 33]. The results showed that HR was significantly favored over RFA for survival outcomes and that RFA was significantly favored over HR for complications and LOS [32, 33]. Although the results for RFA vs. HR are directionally aligned to our meta-analysis of MWA vs. HR, results generally appeared to be indirectly better for MWA vs. RFA. For example, 1-year OS and 5-year DFS outcomes are not significantly different between MWA and HR, whereas RFA is significantly worse than HR (Fig. 6a). MWA and RFA were both significantly favored over HR for complications and LOS, to a similar degree (Fig. 6a, b). Therefore, in liver cancer treatment, MWA appears to as good or better than RFA when both are compared with HR.

The strengths of this analysis include assessment of a broad array of oncological, intra- and post-operative outcomes, inclusion of both HCC and liver metastases studies, and sensitivity analyses to assess the effects of alternative methods and study quality. As discussed above, a key limitation of this meta-analysis was that all studies but one, were observational, and potentially exhibited selection bias given the nature of the two interventions and the variable populations studied. Furthermore, although several outcomes were assessed in this study, few studies reported on both pre- and post- operative liver function tests. This would have been interesting to analyze the extent of benefit MWA could have over resection for preserving liver function, and thus appropriateness in patients with liver comorbidities. Finally, although both HCC and liver metastases studies were included, subgroup analyses on primary tumor type could not be completed due to limited data. As more studies become available, such analyses may be achievable.

## Conclusions

In conclusion, MWA can be an effective and safe alternative to HR in patients/tumors that are not as amenable to resection. Survival and recurrence outcomes were either not statistically significant between MWA and HR or significantly improved by HR. MWA exhibited significantly shorter LOS and operative time, less intraoperative blood loss, and fewer complications. MWA is an important treatment, since relatively few patients are resectable, and there is a potential for significant economic benefits. More randomized trials and economic studies should be performed that compare the two treatments.

### Additional files

Additional file 1: PRISMA checklist. Table of preferred reporting items indicating the page on which each item is located. (DOC 67 kb)

Additional file 2: Search Strategies. List of search terms and results used to obtain the studies reviewed for inclusion in the meta-analysis. (DOCX 52 kb)

Additional file 3: Study treatment selection criteria and resectability. Table describing the selection criteria used to assign patients to different treatments and descriptions of whether MWA patients were resectable. (DOCX 31 kb)

Additional file 4: Methodological quality assessment of observational studies using the NOS scale. Table of Newcastle–Ottawa scale assessments of the included observational studies. (XLSX 10 kb)

Additional file 5: Forest plot of random effects meta-analysis results for 1-year OS (*P* = 0.409). Forest plot of secondary outcome data. Abbreviations: OS, overall survival (PDF 3082 kb)

Additional file 6: Forest plot of random effects meta-analysis results for 3-year OS (*P* = 0.03). Forest plot of secondary outcome data. Abbreviations: OS, overall survival (PDF 3075 kb)

Additional file 7: Forest plot of random effects meta-analysis results for 5-year OS (*P* = 0.01). Forest plot of secondary outcome data. Abbreviations: OS, overall survival (PDF 2537 kb)

Additional file 8: Forest plot of random effects meta-analysis results for IDL (*P* = 0.474). Forest plot of secondary outcome data. Abbreviations: IDL, intrahepatic de novo lesions (PDF 2383 kb)

Additional file 9: Forest plot of random effects meta-analysis results for EHM (*P* = 0.659). Forest plot of secondary outcome data. Abbreviations: EHM, extrahepatic metastasis (PDF 1517 kb)

Additional file 10: Forest plot of random effects meta-analysis results for operative time (*P* < 0.001). Forest plot of secondary outcome data. (PDF 2454 kb)

Additional file 11: Forest plot of random effects meta-analysis results for intraoperative blood loss (*P* = 0.006). Forest plot of secondary outcome data. (PDF 2046 kb)

Additional file 12: Forest plot of random effects meta-analysis results for LOS (*P* < 0.001). Forest plot of secondary outcome data. Abbreviations: LOS, length of stay (PDF 2418 kb)

Additional file 13: Forest plot of random effects meta-analysis results for 1-year DFS (*P* = 0.085). Forest plot of secondary outcome data. Abbreviations: DFS, disease-free survival (PDF 2422 kb)

Additional file 14: Forest plot of random effects meta-analysis results for 3-year DFS (*P* = 0.009). Forest plot of secondary outcome data. Abbreviations: DFS, disease-free survival (PDF 2412 kb)

Additional file 15: Forest plot of random effects meta-analysis results for 5-year DFS (*P* = 0.284). Forest plot of secondary outcome data. Abbreviations: DFS, disease-free survival (PDF 2391 kb)

Additional file 16: Forest plot of random effects meta-analysis results for overall complications (*P* < 0.001). Forest plot of secondary outcome data. (PDF 2903 kb)

Additional file 17: Forest plot of random effects meta-analysis results for major complications (*P* = 0.002). Forest plot of secondary outcome data. (PDF 1739 kb)

Additional file 18: Forest plot of random effects meta-analysis results for minor complications (*P* = 0.024). Forest plot of secondary outcome data. (PDF 1674 kb)

Additional file 19: Funnel plots assessing publication bias. Funnel plots for secondary outcome publication bias assessment. (a) 1-year OS (*n =* 10), (b) 3-year OS (*n =* 10), (c) 5-year OS (*n =* 9), and (d) overall complications (*n =* 8). Red dots indicate the RCT. Abbreviations: OS, overall survival; RCT, randomized controlled trial. (PDF 1214 kb)

## Figures and Tables

**Fig. 7:**
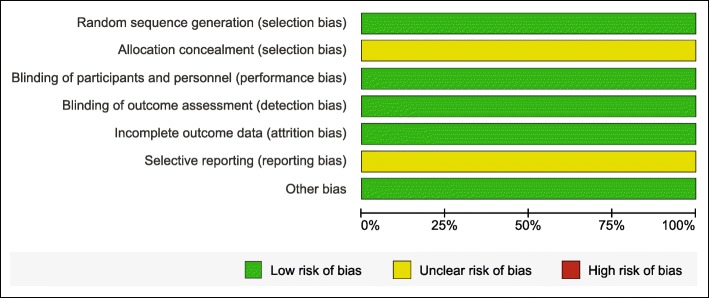
Quality assessment of the Xu 2015 RCT

## Data Availability

All data generated or analyzed during this study are included in this published article [and its supplementary information files].

## Appendix

### Methodological quality assessment of the RCT using the Cochrane RoB tool

**Table 7:** Quality assessment of Xu 2015 using the Cochrane risk of bias tool

Study	Random Sequence Generation	Allocation Concealment	Blinding of participants and personnel	Blinding of outcome assessment	Incomplete outcome data	Selective reporting	Other bias
Xu, 2015	Low	Unclear	Low	Low	Low	Unclear	Low

## References

[1] American Cancer Society. Cancer Facts & Figures 2018 [Available from: https://www.cancer.org/research/cancer-facts-statistics/all-cancer-facts-figures/cancer-facts-figures-2018.html.

[2] Xu J (2018). Trends in liver cancer mortality among adults aged 25 and over in the United States, 2000–2016.

[3] Stattner S, Jones RP, Yip VS, Buchanan K, Poston GJ, Malik HZ (2013). Microwave ablation with or without resection for colorectal liver metastases. Eur J Surg Oncol..

[4] Tanaka K, Shimada H, Nagano Y, Endo I, Sekido H, Togo S (2006). Outcome after hepatic resection versus combined resection and microwave ablation for multiple bilobar colorectal metastases to the liver. Surgery..

[5] Kim C (2018). Understanding the nuances of microwave ablation for more accurate post-treatment assessment. Future Oncol..

[6] Zhang EL, Yang F, Wu ZB, Yue CS, He TY, Li KY (2016). Therapeutic efficacy of percutaneous microwave coagulation versus liver resection for single hepatocellular carcinoma </=3 cm with Child-Pugh A cirrhosis. Eur J Surg Oncol..

[7] Ryu T, Takami Y, Wada Y, Tateishi M, Matsushima H, Yoshitomi M, et al. Oncological outcomes after hepatic resection and/or surgical microwave ablation for liver metastasis from gastric cancer. Asian J Surg. 2017. 10.1016/j.asjsur.2017.09.005 29254868

[8] Luo W, Zhang Y, He G, Yu M, Zheng M, Liu L (2017). Effects of radiofrequency ablation versus other ablating techniques on hepatocellular carcinomas: a systematic review and meta-analysis. World J Surg Oncol..

[9] Huo YR, Eslick GD (2015). Microwave ablation compared to radiofrequency ablation for hepatic lesions: a meta-analysis. J Vasc Interv Radiol..

[10] Zhang M, Ma H, Zhang J, He L, Ye X, Li X (2017). Comparison of microwave ablation and hepatic resection for hepatocellular carcinoma: a meta-analysis. Onco Targets Ther..

[11] Imura S, Shimada M, Utsunomiya T, Morine Y, Ikemoto T, Mori H (2012). Ultrasound-guided microwave coagulation assists anatomical hepatic resection. Surg Today..

[12] Tan K, Du X, Yin J, Dong R, Zang L, Yang T (2014). Microwave tissue coagulation technique in anatomical liver resection. Biomed Rep..

[13] Philips P, Scoggins CR, Rostas JK, McMasters KM, Martin RC (2017). Safety and advantages of combined resection and microwave ablation in patients with bilobar hepatic malignancies. Int J Hyperthermia..

[14] Song P, Sheng L, Sun Y, An Y, Guo Y, Zhang Y (2017). The clinical utility and outcomes of microwave ablation for colorectal cancer liver metastases. Oncotarget..

[15] Zhang QB, Zhang XG, Jiang RD, Hu CX, Sun D, Ran L (2017). Microwave ablation versus hepatic resection for the treatment of hepatocellular carcinoma and oesophageal variceal bleeding in cirrhotic patients. Int J Hyperthermia..

[16] Chen ZB, Qin F, Ye Z, Shen SQ, Li W, Ding YM, et al. Microwave-assisted liver resection vs. clamp crushing liver resection in cirrhosis patients with hepatocellular carcinoma. Int J Hyperthermia. 2018:1–8. 10.1080/02656736.2018.1429678 29353503

[17] Chong CCN, Lee KF, Chu CM, Chan AWH, Wong J, Chan SL (2018). Microwave ablation provides better survival than liver resection for hepatocellular carcinoma in patients with borderline liver function: application of ALBI score to patient selection. HPB (Oxford)..

[18] Moher D, Liberati A, Tetzlaff J, Altman DG, Group P (2009). Preferred reporting items for systematic reviews and meta-analyses: the PRISMA statement. PLoS Med.

[19] McGowan J, Sampson M, Salzwedel DM, Cogo E, Foerster V, Lefebvre C (2016). PRESS Peer Review of Electronic Search Strategies: 2015 Guideline Statement. J Clin Epidemiol..

[20] Higgins J, Green S, (editors). Cochrane Handbook for Systematic Reviews of Interventions Version 5.1.0 [updated March 2011. Available from: http://handbook.cochrane.org.

[21] Wells G, Shea B, O'Connell D, Peterson J, Welch V, Losos M, et al. The Newcastle–Ottawa scale (NOS) for assessing the quality of nonrandomized studies in metaanalysis [updated 2014. Available from: http://www.ohri.ca/programs/clinical_epidemiology/oxford.asp.

[22] Bruix J, Reig M, Sherman M (2016). Evidence-based diagnosis, staging, and treatment of patients with hepatocellular carcinoma. Gastroenterology..

[23] Sterne JA, Sutton AJ, Ioannidis JP, Terrin N, Jones DR, Lau J (2011). Recommendations for examining and interpreting funnel plot asymmetry in meta-analyses of randomised controlled trials. BMJ..

[24] Xu J, Zhao Y (2015). Comparison of percutaneous microwave ablation and laparoscopic resection in the prognosis of liver cancer. Int J Clin Exp Pathol..

[25] Wang ZL, Liang P, Dong BW, Yu XL, Yu DJ (2008). Prognostic factors and recurrence of small hepatocellular carcinoma after hepatic resection or microwave ablation: a retrospective study. J Gastrointest Surg..

[26] Takami Y, Ryu T, Wada Y, Saitsu H (2013). Evaluation of intraoperative microwave coagulo-necrotic therapy (MCN) for hepatocellular carcinoma: a single center experience of 719 consecutive cases. J Hepatobiliary Pancreat Sci..

[27] Shi J, Sun Q, Wang Y, Jing X, Ding J, Yuan Q (2014). Comparison of microwave ablation and surgical resection for treatment of hepatocellular carcinomas conforming to Milan criteria. J Gastroenterol Hepatol..

[28] Li W, Zhou X, Huang Z, Zhang K, Luo X, Zhong J (2017). Short-term and long-term outcomes of laparoscopic hepatectomy, microwave ablation, and open hepatectomy for small hepatocellular carcinoma: a 5-year experience in a single center. Hepatol Res..

[29] Daskalaki D, Gonzalez-Heredia R, Brown M, Bianco FM, Tzvetanov I, Davis M (2017). Financial impact of the robotic approach in liver surgery: a comparative study of clinical outcomes and costs between the robotic and open technique in a single institution. J Laparoendosc Adv Surg Tech A..

[30] Puijk RS, Ruarus AH, Vroomen L, van Tilborg A, Scheffer HJ, Nielsen K (2018). Colorectal liver metastases: surgery versus thermal ablation (COLLISION) - a phase III single-blind prospective randomized controlled trial. BMC Cancer..

[31] Cucchetti A, Piscaglia F, Cescon M, Colecchia A, Ercolani G, Bolondi L (2013). Cost-effectiveness of hepatic resection versus percutaneous radiofrequency ablation for early hepatocellular carcinoma. J Hepatol..

[32] Feng Q, Chi Y, Liu Y, Zhang L, Liu Q (2015). Efficacy and safety of percutaneous radiofrequency ablation versus surgical resection for small hepatocellular carcinoma: a meta-analysis of 23 studies. J Cancer Res Clin Oncol..

[33] Xu Q, Kobayashi S, Ye X, Meng X (2014). Comparison of hepatic resection and radiofrequency ablation for small hepatocellular carcinoma: a meta-analysis of 16,103 patients. Sci Rep..

