# Supplementary material for: Microwave ablation compared with hepatic resection for the treatment of hepatocellular carcinoma and liver metastases: a systematic review and meta-analysis
Source: World J Surg Oncol. 2019 Jun 10;17:98. doi: 10.1186/s12957-019-1632-6 (PMC6558848; doi:10.1186/s12957-019-1632-6)
Supplement: Supplementary file 2 — Search Strategies. List of search terms and results used to obtain the studies reviewed for inclusion in the meta-analysis. (DOCX 52 kb) [file 12957_2019_1632_MOESM2_ESM.docx]

**Search strategies**

**Microwave Ablation**

**Final Strategies**

2017 Oct 29

Ovid Multifile

ALL CANCERS

Database: EBM Reviews - Cochrane Central Register of Controlled Trials <September 2017>, EBM Reviews - Cochrane Database of Systematic Reviews <2005 to October 25, 2017>, Embase <1988 to 2017 Week 44>, Ovid MEDLINE(R) Epub Ahead of Print, In-Process & Other Non-Indexed Citations, Ovid MEDLINE(R) Daily and Ovid MEDLINE(R) <1946 to Present>

Search Strategy:

--------------------------------------------------------------------------------

1 exp neoplasms/ (6548332)

2 (hepatoma* or hepatoblastoma* or hepato-blastoma*).tw,kf. (58693)

3 (neoplas* or cancer* or tumour* or tumor* or carcinoma* or malignan* or metasta* or oncolog*).tw,kf. (6584383)

4 (adenoma? or adenocarcinoma? or adeno-carcinoma? or blastoma? or carcinosarcoma? or carcino-sarcoma? or leukemia? or leukaemia? or lymphoma? or melanoma? or mesenchymoma? or mesothelioma? or sarcoma? or thymoma?).tw,kf. (1611251)

5 HCC.tw,kf. (113183)

6 or/1-5 [CANCER] (8457182)

7 (microwave* adj3 (therap* or treatment*)).tw,kf. (3780)

8 MW ablation*.tw,kf. (291)

9 MW coagulation*.tw,kf. (3)

10 (MW adj (electrocoagulation* or electro-coagulation*)).tw,kf. (0)

11 (MW adj (thermocoagulation* or thermo-coagulation*)).tw,kf. (0)

12 (MW adj (electrocauter* or electro-cauter*)).tw,kf. (0)

13 (MW adj (thermal therap* or thermotherap* or thermo-therap*)).tw,kf. (6)

14 MWA.tw,kw. (1670)

15 (PMCT or PMCTs).tw,kf. (810)

16 (PMAT or PMATs).tw,kf. (248)

17 (RALTCT or RALTCTs).tw,kf. (2)

18 (MCT or MCTs).tw,kf. (13985)

19 or/7-18 (20281)

20 Microwaves/ (32107)

21 microwave*.tw,kf. (61028)

22 (extremely high frequency adj (radiowave* or radio wave* or wave*)).tw,kf. (0)

23 (EHF adj (radiowave* or radio wave* or wave*)).tw,kf. (12)

24 ((ultrahigh or ultra-high) adj frequency adj (radiowave* or radio wave* or wave*)).tw,kf. (25)

25 or/20-24 [MICROWAVES] (66138)

26 exp Ablation Techniques/ (129498)

27 ablat*.tw,kf. (234249)

28 thermal heat*.tw,kf. (847)

29 (thermal therap* or thermotherap* or thermo-therap*).tw,kf. (7877)

30 Electrocoagulation/ (17638)

31 coagulation*.tw,kf. (194496)

32 (electrocoagulation* or electro-coagulation*).tw,kf. (6356)

33 (thermocoagulation* or thermo-coagulation*).tw,kf. (2050)

34 (electrocauter* or electro-cauter*).tw,kf. (8280)

35 or/26-34 [ABLATION/COAGULATION] (519911)

36 25 and 35 (7570)

37 (Acculis* or Amica* or AMICA-GEM* or Avecure* or Certus* or Emblation* or Emprint* or FORSEA* or MicrothermX* or MSYS245 or NEUWAVE* or Solero* or TATO or TATOPro*).tw,kf. (1474)

38 (Evident* and Covidien).tw,kf. (45)

39 37 or 38 [MICROWAVE ABLATION DEVICES/SYSTEMS] (1518)

40 19 or 36 or 39 [MICROWAVE ABLATION/COAGULATION] (26406)

41 6 and 40 [MICROWAVE ABLATION IN CANCER] (8781)

42 exp Animals/ not (exp Animals/ and Humans/) (13742529)

43 41 not 42 [ANIMAL-ONLY REMOVED] (5902)

44 (comment or editorial or interview or news or newspaper article).pt. (1797220)

45 (letter not (letter and randomized controlled trial)).pt. (1890383)

46 43 not (44 or 45) [OPINION PIECES REMOVED] (5791)

47 limit 46 to systematic reviews [Limit not valid in CCTR,CDSR,Embase; records were retained] (2792)

48 meta analysis.pt. (92808)

49 exp meta-analysis as topic/ (55201)

50 (meta-analy* or metanaly* or metaanaly* or met analy* or integrative research or integrative review* or integrative overview* or research integration or research overview* or collaborative review*).tw,kf. (308371)

51 (systematic review* or systematic overview* or evidence-based review* or evidence-based overview* or (evidence adj3 (review* or overview*)) or meta-review* or meta-overview* or meta-synthes* or rapid review* or "review of reviews" or technology assessment* or HTA or HTAs).tw,kf. (356845)

52 exp Technology assessment, biomedical/ (22439)

53 (cochrane or health technology assessment or evidence report).jw. (46132)

54 ((indirect* or mixed or multi-treatment*) adj2 compar*).tw,kf. (11524)

55 ((network* or network-based) adj (MA or MAs)).tw,kf. (18)

56 or/48-55 (639047)

57 46 and 56 (164)

58 47 or 57 [SYSTEMATIC REVIEWS/META-ANALYSES] (2807)

59 (controlled clinical trial or randomized controlled trial).pt. (1108409)

60 "Clinical Trials as Topic".sh. (228911)

61 exp Randomized Controlled Trials as Topic/ (270316)

62 (randomi#ed or randomi#ation* or randomly or RCT$1 or placebo*).tw,kf. (2598052)

63 ((singl* or doubl* or trebl* or tripl*) adj (mask* or blind* or dumm*)).tw,kf. (544046)

64 trial.ti. (610226)

65 or/59-64 (3362039)

66 46 and 65 [RCTS] (601)

67 58 or 66 [SRs/MAs, RCTs] (3009)

68 67 use ppez [MEDLINE RECORDS] (300)

69 exp neoplasm/ (6548332)

70 (hepatoma* or hepatoblastoma* or hepato-blastoma*).tw,kw. (59260)

71 (neoplas* or cancer* or tumour* or tumor* or carcinoma* or malignan* or metasta* or oncolog*).tw,kw. (6586761)

72 (adenoma? or adenocarcinoma? or adeno-carcinoma? or blastoma? or carcinosarcoma? or carcino-sarcoma? or leukemia? or leukaemia? or lymphoma? or melanoma? or mesenchymoma? or mesothelioma? or sarcoma? or thymoma?).tw,kw. (1617620)

73 HCC.tw,kw. (113658)

74 or/69-73 [LIVER CANCER] (8477025)

75 exp microwave thermotherapy/ (4237)

76 (microwave* adj3 (therap* or treatment*)).tw,kw. (3838)

77 MW ablation*.tw,kw. (292)

78 MW coagulation*.tw,kw. (3)

79 (MW adj (electrocoagulation* or electro-coagulation*)).tw,kw. (0)

80 (MW adj (thermocoagulation* or thermo-coagulation*)).tw,kw. (0)

81 (MW adj (electrocauter* or electro-cauter*)).tw,kw. (0)

82 (MW adj (thermal therap* or thermotherap* or thermo-therap*)).tw,kw. (6)

83 MWA.tw,kw. (1670)

84 (PMCT or PMCTs).tw,kw. (822)

85 (PMAT or PMATs).tw,kw. (248)

86 (RALTCT or RALTCTs).tw,kw. (2)

87 (MCT or MCTs).tw,kw. (14061)

88 or/75-87 (24228)

89 microwave radiation/ (17072)

90 microwave*.tw,kw. (61798)

91 (extremely high frequency adj (radiowave* or radio wave* or wave*)).tw,kw. (0)

92 (EHF adj (radiowave* or radio wave* or wave*)).tw,kw. (16)

93 ((ultrahigh or ultra-high) adj frequency adj (radiowave* or radio wave* or wave*)).tw,kw. (25)

94 or/89-93 [MICROWAVES] (64242)

95 ablation device/ (372)

96 ablation therapy/ (13635)

97 thermal ablation delivery device/ (47)

98 ablat*.tw,kw. (236509)

99 thermal heat*.tw,kw. (856)

100 (thermal therap* or thermotherap* or thermo-therap*).tw,kw. (8177)

101 electrocoagulation/ (17638)

102 coagulation*.tw,kw. (194133)

103 (electrocoagulation* or electro-coagulation*).tw,kw. (6567)

104 (thermocoagulation* or thermo-coagulation*).tw,kw. (2092)

105 (electrocauter* or electro-cauter*).tw,kw. (8393)

106 or/95-105 [ABLATION/COAGULATION] (456758)

107 94 and 106 (7398)

108 (Acculis* or Amica* or AMICA-GEM* or Avecure* or Certus* or Emblation* or Emprint* or FORSEA* or MicrothermX* or MSYS245 or NEUWAVE* or Solero* or TATO or TATOPro*).tw,kf,dv. (1634)

109 Evident.dv. (24)

110 (Evident* and Covidien).tw,kf. (45)

111 or/108-110 [MICROWAVE ABLATION DEVICES/SYSTEMS] (1695)

112 88 or 107 or 111 [MICROWAVE ABLATION/COAGULATION] (29769)

113 74 and 112 [MICROWAVE ABLATION IN LIVER CANCER] (9758)

114 exp animal experimentation/ or exp animal model/ or exp animal experiment/ or nonhuman/ or exp vertebrate/ (42820802)

115 exp human/ or exp human experimentation/ or exp human experiment/ (34420509)

116 114 not 115 (8401488)

117 113 not 116 [ANIMAL-ONLY REMOVED] (8653)

118 editorial.pt. (987409)

119 letter.pt. not (letter.pt. and randomized controlled trial/) (1890417)

120 117 not (118 or 119) [OPINION PIECES REMOVED] (8483)

121 meta-analysis/ (228303)

122 "systematic review"/ (153364)

123 (meta-analy* or metanaly* or metaanaly* or met analy* or integrative research or integrative review* or integrative overview* or research integration or research overview* or collaborative review*).tw,kw. (311557)

124 (systematic review* or systematic overview* or evidence-based review* or evidence-based overview* or (evidence adj3 (review* or overview*)) or meta-review* or meta-overview* or meta-synthes* or rapid review* or "review of reviews" or technology assessment* or HTA or HTAs).tw,kw. (360537)

125 biomedical technology assessment/ (21300)

126 (cochrane or health technology assessment or evidence report).jw. (46132)

127 ((indirect* or mixed or multi-treatment*) adj2 compar*).tw,kw. (11560)

128 ((network* or network-based) adj (MA or MAs)).tw,kw. (18)

129 or/121-128 (668041)

130 120 and 129 [SYSTEMATIC REVIEWS/META-ANALYSES] (258)

131 randomized controlled trial/ or controlled clinical trial/ (1224565)

132 exp "clinical trial (topic)"/ (256771)

133 (randomi#ed or randomi#ation* or randomly or RCT$1 or placebo*).tw,kw. (2610952)

134 ((singl* or doubl* or trebl* or tripl*) adj (mask* or blind* or dumm*)).tw,kw. (554873)

135 trial.ti. (610226)

136 or/131-135 (3367162)

137 120 and 136 [RCTs] (992)

138 130 or 137 [REVIEWS, RCTs] (1104)

139 138 use emed [EMBASE RECORDS] (547)

140 exp neoplasms/ (6548332)

141 (hepatoma* or hepatoblastoma* or hepato-blastoma*).ti,ab,kw. (59239)

142 (neoplas* or cancer* or tumour* or tumor* or carcinoma* or malignan* or metasta* or oncolog*).ti,ab,kw. (6584289)

143 (adenoma? or adenocarcinoma? or adeno-carcinoma? or blastoma? or carcinosarcoma? or carcino-sarcoma? or leukemia? or leukaemia? or lymphoma? or melanoma? or mesenchymoma? or mesothelioma? or sarcoma? or thymoma?).ti,ab,kw. (1617008)

144 HCC.ti,ab,kw. (113634)

145 or/140-144 [CANCER] (8474692)

146 (microwave* adj3 (therap* or treatment*)).ti,ab,kw. (3825)

147 MW ablation*.ti,ab,kw. (292)

148 MW coagulation*.ti,ab,kw. (3)

149 (MW adj (electrocoagulation* or electro-coagulation*)).ti,ab,kw. (0)

150 (MW adj (thermocoagulation* or thermo-coagulation*)).ti,ab,kw. (0)

151 (MW adj (electrocauter* or electro-cauter*)).ti,ab,kw. (0)

152 (MW adj (thermal therap* or thermotherap* or thermo-therap*)).ti,ab,kw. (6)

153 MWA.ti,ab,kw. (1670)

154 (PMCT or PMCTs).ti,ab,kw. (822)

155 (PMAT or PMATs).ti,ab,kw. (248)

156 (RALTCT or RALTCTs).ti,ab,kw. (2)

157 (MCT or MCTs).ti,ab,kw. (14007)

158 or/146-157 (20346)

159 Microwaves/ (32107)

160 microwave*.ti,ab,kw. (61760)

161 (extremely high frequency adj (radiowave* or radio wave* or wave*)).ti,ab,kw. (0)

162 (EHF adj (radiowave* or radio wave* or wave*)).ti,ab,kw. (15)

163 ((ultrahigh or ultra-high) adj frequency adj (radiowave* or radio wave* or wave*)).ti,ab,kw. (24)

164 or/159-163 [MICROWAVES] (66730)

165 exp Ablation Techniques/ (129498)

166 ablat*.ti,ab,kw. (236328)

167 thermal heat*.ti,ab,kw. (853)

168 (thermal therap* or thermotherap* or thermo-therap*).ti,ab,kw. (8141)

169 Electrocoagulation/ (17638)

170 coagulation*.ti,ab,kw. (193715)

171 (electrocoagulation* or electro-coagulation*).ti,ab,kw. (6542)

172 (thermocoagulation* or thermo-coagulation*).ti,ab,kw. (2082)

173 (electrocauter* or electro-cauter*).ti,ab,kw. (8331)

174 or/165-173 [ABLATION/COAGULATION] (521407)

175 164 and 174 (7652)

176 (Acculis* or Amica* or AMICA-GEM* or Avecure* or Certus* or Emblation* or Emprint* or FORSEA* or MicrothermX* or MSYS245 or NEUWAVE* or Solero* or TATO or TATOPro*).ti,ab,kw. (1227)

177 (Evident* and Covidien).ti,ab,kw. (36)

178 176 or 177 [MICROWAVE ABLATION DEVICES/SYSTEMS] (1262)

179 158 or 175 or 178 [MICROWAVE ABLATION/COAGULATION] (26266)

180 145 and 179 [MICROWAVE ABLATION IN CANCER] (8835)

181 180 use cctr,coch [CENTRAL, DSR RECORDS] (182)

182 68 or 139 or 181 [ALL DATABASES] (1029)

**183 remove duplicates from 182 (654) [TOTAL UNIQUE RECORDS]**

184 183 use ppez [MEDLINE UNIQUE RECORDS] (264)

185 183 use emed [EMBASE UNIQUE RECORDS] (342)

186 183 use cctr [CENTRAL RECORDS] (42)

187 183 use coch [DSR UNIQUE RECORDS] (6)

**Microwave Ablation – Non-RCTs/Observational Studies**

Ovid Multifile

2017 Nov 24

**Liver Cancer**

Database: Embase <1988 to 2017 Week 47>, Ovid MEDLINE(R) Epub Ahead of Print, In-Process & Other Non-Indexed Citations, Ovid MEDLINE(R) Daily and Ovid MEDLINE(R) <1946 to Present>

Search Strategy:

--------------------------------------------------------------------------------

1 exp Liver Neoplasms/ (367294)

2 (hepatoma* or hepatoblastoma* or hepato-blastoma*).tw,kf. (58994)

3 ((neoplas* or cancer* or tumour* or tumor* or carcinoma* or malignan* or metasta* or oncolog*) adj3 (liver* or hepat*)).tw,kf. (348272)

4 ((adenoma? or adenocarcinoma? or adeno-carcinoma? or blastoma? or carcinosarcoma? or carcino-sarcoma? or leukemia? or leukaemia? or lymphoma? or melanoma? or mesenchymoma? or mesothelioma? or sarcoma? or thymoma?) adj3 (liver* or hepat*)).tw,kf. (17103)

5 HCC.tw,kf. (113949)

6 or/1-5 [LIVER CANCER] (497507)

7 (microwave* adj3 (therap* or treatment*)).tw,kf. (3648)

8 MW ablation*.tw,kf. (282)

9 MW coagulation*.tw,kf. (3)

10 (MW adj (electrocoagulation* or electro-coagulation*)).tw,kf. (0)

11 (MW adj (thermocoagulation* or thermo-coagulation*)).tw,kf. (0)

12 (MW adj (electrocauter* or electro-cauter*)).tw,kf. (0)

13 (MW adj (thermal therap* or thermotherap* or thermo-therap*)).tw,kf. (6)

14 MWA.tw,kf. (1652)

15 (PMCT or PMCTs).tw,kf. (807)

16 (PMAT or PMATs).tw,kf. (246)

17 (RALTCT or RALTCTs).tw,kf. (2)

18 (MCT or MCTs).tw,kf. (13527)

19 or/7-18 (19666)

20 Microwaves/ (32183)

21 microwave*.tw,kf. (61070)

22 (extremely high frequency adj (radiowave* or radio wave* or wave*)).tw,kf. (0)

23 (EHF adj (radiowave* or radio wave* or wave*)).tw,kf. (11)

24 ((ultrahigh or ultra-high) adj frequency adj (radiowave* or radio wave* or wave*)).tw,kf. (24)

25 or/20-24 [MICROWAVES] (66163)

26 exp Ablation Techniques/ (127256)

27 ablat*.tw,kf. (232699)

28 thermal heat*.tw,kf. (831)

29 (thermal therap* or thermotherap* or thermo-therap*).tw,kf. (7650)

30 Electrocoagulation/ (17172)

31 coagulation*.tw,kf. (190845)

32 (electrocoagulation* or electro-coagulation*).tw,kf. (6211)

33 (thermocoagulation* or thermo-coagulation*).tw,kf. (1959)

34 (electrocauter* or electro-cauter*).tw,kf. (7889)

35 or/26-34 [ABLATION/COAGULATION] (512271)

36 25 and 35 (7411)

37 (Acculis* or Amica* or AMICA-GEM* or Avecure* or Certus* or Emblation* or Emprint* or FORSEA* or MicrothermX* or MSYS245 or NEUWAVE* or Solero* or TATO or TATOPro*).tw,kf. (1441)

38 (Evident* and Covidien).tw,kf. (38)

39 37 or 38 [MICROWAVE ABLATION DEVICES/SYSTEMS] (1478)

40 19 or 36 or 39 [MICROWAVE ABLATION/COAGULATION] (25665)

41 6 and 40 [MICROWAVE ABLATION IN LIVER CANCER] (2968)

42 exp Animals/ not (exp Animals/ and Humans/) (13815981)

43 41 not 42 [ANIMAL-ONLY REMOVED] (2141)

44 (comment or editorial or interview or news or newspaper article).pt. (1811310)

45 (letter not (letter and randomized controlled trial)).pt. (1902526)

46 43 not (44 or 45) [OPINION PIECES REMOVED] (2099)

47 controlled clinical trial.pt. (100426)

48 Controlled Clinical Trial/ or Controlled Clinical Trials as Topic/ (566284)

49 (control* adj2 trial*).tw,kf. (510234)

50 Non-Randomized Controlled Trials as Topic/ (9787)

51 (nonrandom* or non-random* or quasi-random* or quasi-experiment*).tw,kf. (104158)

52 (nRCT or nRCTs or non-RCT?).tw,kf. (1529)

53 Controlled Before-After Studies/ (102794)

54 (control* adj3 ("before and after" or "before after")).tw,kf. (8251)

55 Interrupted Time Series Analysis/ (94733)

56 time series.tw,kf. (51316)

57 (pre- adj3 post-).tw,kf. (176317)

58 (pretest adj3 posttest).tw,kf. (9181)

59 Historically Controlled Study/ (113070)

60 (control* adj2 stud$3).tw,kf. (466388)

61 Control Groups/ (113596)

62 (control* adj2 group$1).tw,kf. (1017517)

63 trial.ti. (418799)

64 or/47-63 (2820009)

65 46 and 64 [NON-RCTS] (175)

66 exp Cohort Studies/ (2202031)

67 cohort?.tw,kf. (1199921)

68 Retrospective Studies/ (1081446)

69 (longitudinal or prospective or retrospective).tw,kf. (2576412)

70 ((followup or follow-up) adj (study or studies)).tw,kf. (98667)

71 Observational study.pt. (48871)

72 (observation$2 adj (study or studies)).tw,kf. (205711)

73 ((population or population-based) adj (study or studies or analys#s)).tw,kf. (34353)

74 ((multidimensional or multi-dimensional) adj (study or studies)).tw,kf. (209)

75 Comparative Study.pt. (1936005)

76 ((comparative or comparison) adj (study or studies)).tw,kf. (200618)

77 exp Case-Control Studies/ (1114532)

78 ((case-control* or case-based or case-comparison) adj (study or studies)).tw,kf. (208928)

79 or/66-78 (6669145)

80 46 and 79 [OBSERVATIONAL STUDIES] (570)

81 65 or 80 [NON-RCTS, OBSERVATIONAL STUDIES] (671)

82 81 use ppez [MEDLINE RECORDS] (434)

83 exp liver cancer/ (346160)

84 (hepatoma* or hepatoblastoma* or hepato-blastoma*).tw,kw. (59563)

85 ((neoplas* or cancer* or tumour* or tumor* or carcinoma* or malignan* or metasta* or oncolog*) adj3 (liver* or hepat*)).tw,kw. (351453)

86 ((adenoma? or adenocarcinoma? or adeno-carcinoma? or blastoma? or carcinosarcoma? or carcino-sarcoma? or leukemia? or leukaemia? or lymphoma? or melanoma? or mesenchymoma? or mesothelioma? or sarcoma? or thymoma?) adj3 (liver* or hepat*)).tw,kw. (17655)

87 HCC.tw,kw. (114424)

88 or/83-87 [LIVER CANCER] (492543)

89 exp microwave thermotherapy/ (3964)

90 (microwave* adj3 (therap* or treatment*)).tw,kw. (3689)

91 MW ablation*.tw,kw. (283)

92 MW coagulation*.tw,kw. (3)

93 (MW adj (electrocoagulation* or electro-coagulation*)).tw,kw. (0)

94 (MW adj (thermocoagulation* or thermo-coagulation*)).tw,kw. (0)

95 (MW adj (electrocauter* or electro-cauter*)).tw,kw. (0)

96 (MW adj (thermal therap* or thermotherap* or thermo-therap*)).tw,kw. (6)

97 MWA.tw,kw. (1653)

98 (PMCT or PMCTs).tw,kw. (819)

99 (PMAT or PMATs).tw,kw. (246)

100 (RALTCT or RALTCTs).tw,kw. (2)

101 (MCT or MCTs).tw,kw. (13599)

102 or/89-101 (23323)

103 microwave radiation/ (17130)

104 microwave*.tw,kw. (61837)

105 (extremely high frequency adj (radiowave* or radio wave* or wave*)).tw,kw. (0)

106 (EHF adj (radiowave* or radio wave* or wave*)).tw,kw. (11)

107 ((ultrahigh or ultra-high) adj frequency adj (radiowave* or radio wave* or wave*)).tw,kw. (24)

108 or/103-107 [MICROWAVES] (64283)

109 ablation device/ (376)

110 ablation therapy/ (13773)

111 thermal ablation delivery device/ (49)

112 ablat*.tw,kw. (234895)

113 thermal heat*.tw,kw. (840)

114 (thermal therap* or thermotherap* or thermo-therap*).tw,kw. (7904)

115 electrocoagulation/ (17172)

116 coagulation*.tw,kw. (190305)

117 (electrocoagulation* or electro-coagulation*).tw,kw. (6389)

118 (thermocoagulation* or thermo-coagulation*).tw,kw. (2000)

119 (electrocauter* or electro-cauter*).tw,kw. (8000)

120 or/109-119 [ABLATION/COAGULATION] (450547)

121 108 and 120 (7232)

122 (Acculis* or Amica* or AMICA-GEM* or Avecure* or Certus* or Emblation* or Emprint* or FORSEA* or MicrothermX* or MSYS245 or NEUWAVE* or Solero* or TATO or TATOPro*).tw,kf,dv. (1605)

123 Evident.dv. (24)

124 (Evident* and Covidien).tw,kf. (38)

125 or/122-124 [MICROWAVE ABLATION DEVICES/SYSTEMS] (1659)

126 102 or 121 or 125 [MICROWAVE ABLATION/COAGULATION] (28748)

127 88 and 126 [MICROWAVE ABLATION IN LIVER CANCER] (3025)

128 exp animal experimentation/ or exp animal model/ or exp animal experiment/ or nonhuman/ or exp vertebrate/ (42676872)

129 exp human/ or exp human experimentation/ or exp human experiment/ (34212875)

130 128 not 129 (8465195)

131 127 not 130 [ANIMAL-ONLY REMOVED] (2866)

132 editorial.pt. (994395)

133 letter.pt. not (letter.pt. and randomized controlled trial/) (1897936)

134 131 not (132 or 133) [OPINION PIECES REMOVED] (2803)

135 exp controlled clinical trial/ (1239314)

136 exp "controlled clinical trial (topic)"/ (146336)

137 (control* adj2 trial*).tw,kw. (514171)

138 (nonrandom* or non-random* or quasi-random* or quasi-experiment*).tw,kw. (104332)

139 (nRCT or nRCTs or non-RCT$1).tw,kw. (1529)

140 (control* adj3 ("before and after" or "before after")).tw,kw. (8256)

141 time series analysis/ (20466)

142 time series.tw,kw. (52021)

143 pretest posttest control group design/ (332)

144 (pre- adj3 post-).tw,kw. (176342)

145 (pretest adj3 posttest).tw,kw. (9185)

146 controlled study/ (5775473)

147 (control* adj2 stud$3).tw,kw. (467841)

148 control group/ (113596)

149 (control* adj2 group$1).tw,kw. (1017262)

150 trial.ti. (418799)

151 or/135-150 (7933882)

152 134 and 151 [NON-RCTS] (546)

153 cohort analysis/ (574262)

154 cohort?.tw,kw. (1201988)

155 retrospective study/ (1318071)

156 longitudinal study/ (229072)

157 prospective study/ (921727)

158 (longitudinal or prospective or retrospective).tw,kw. (2581234)

159 follow up/ (1201915)

160 ((followup or follow-up) adj (study or studies)).tw,kw. (100421)

161 observational study/ (179871)

162 (observation$2 adj (study or studies)).tw,kw. (206275)

163 population research/ (87990)

164 ((population or population-based) adj (study or studies or analys#s)).tw,kw. (42728)

165 ((multidimensional or multi-dimensional) adj (study or studies)).tw,kw. (210)

166 exp comparative study/ (2922216)

167 ((comparative or comparison) adj (study or studies)).tw,kw. (198692)

168 exp case control study/ (1114532)

169 ((case-control* or case-based or case-comparison) adj (study or studies)).tw,kw. (210348)

170 or/153-169 (8074332)

171 134 and 170 [OBSERVATIONAL STUDIES] (1116)

172 152 or 171 [NON-RCTS, OBSERVATIONAL STUDIES] (1360)

173 172 use emed [EMBASE RECORDS] (986)

174 82 or 173 [BOTH DATABASES] (1420)

**175 remove duplicates from 174 (1037)**

176 175 use ppez [MEDLINE UNIQUE RECORDS] (378)

177 175 use emed [EMBASE UNIQUE RECORDS] (659)

**Microwave Ablation – Liver Cancer**

2018 Mar 16 Update

*RCTs, Reviews*

MEDLINE

Database: Ovid MEDLINE(R) Epub Ahead of Print, In-Process & Other Non-Indexed Citations, Ovid MEDLINE(R) Daily and Ovid MEDLINE(R) <1946 to Present>

Search Strategy:

--------------------------------------------------------------------------------

1 exp Liver Neoplasms/ (148591)

2 (hepatoma* or hepatoblastoma* or hepato-blastoma*).tw,kf. (30354)

3 ((neoplas* or cancer* or tumour* or tumor* or carcinoma* or malignan* or metasta* or oncolog*) adj3 (liver* or hepat*)).tw,kf. (148939)

4 ((adenoma? or adenocarcinoma? or adeno-carcinoma? or blastoma? or carcinosarcoma? or carcino-sarcoma? or leukemia? or leukaemia? or lymphoma? or melanoma? or mesenchymoma? or mesothelioma? or sarcoma? or thymoma?) adj3 (liver* or hepat*)).tw,kf. (7860)

5 HCC.tw,kf. (43282)

6 or/1-5 [LIVER CANCER] (214883)

7 (microwave* adj3 (therap* or treatment*)).tw,kf. (1656)

8 MW ablation*.tw,kf. (95)

9 MW coagulation*.tw,kf. (1)

10 (MW adj (electrocoagulation* or electro-coagulation*)).tw,kf. (0)

11 (MW adj (thermocoagulation* or thermo-coagulation*)).tw,kf. (0)

12 (MW adj (electrocauter* or electro-cauter*)).tw,kf. (0)

13 (MW adj (thermal therap* or thermotherap* or thermo-therap*)).tw,kf. (3)

14 MWA.tw,kf. (587)

15 (PMCT or PMCTs).tw,kf. (326)

16 (PMAT or PMATs).tw,kf. (89)

17 (RALTCT or RALTCTs).tw,kf. (1)

18 (MCT or MCTs).tw,kf. (5504)

19 or/7-18 (8058)

20 Microwaves/ (15399)

21 microwave*.tw,kf. (30647)

22 (extremely high frequency adj (radiowave* or radio wave* or wave*)).tw,kf. (0)

23 (EHF adj (radiowave* or radio wave* or wave*)).tw,kf. (6)

24 ((ultrahigh or ultra-high) adj frequency adj (radiowave* or radio wave* or wave*)).tw,kf. (15)

25 or/20-24 [MICROWAVES] (33236)

26 exp Ablation Techniques/ (103925)

27 ablat*.tw,kf. (94738)

28 thermal heat*.tw,kf. (394)

29 (thermal therap* or thermotherap* or thermo-therap*).tw,kf. (3410)

30 Electrocoagulation/ (11077)

31 coagulation*.tw,kf. (90180)

32 (electrocoagulation* or electro-coagulation*).tw,kf. (3438)

33 (thermocoagulation* or thermo-coagulation*).tw,kf. (936)

34 (electrocauter* or electro-cauter*).tw,kf. (3318)

35 or/26-34 [ABLATION/COAGULATION] (256508)

36 25 and 35 (3171)

37 (Acculis* or Amica* or AMICA-GEM* or Avecure* or Certus* or Emblation* or Emprint* or FORSEA* or MicrothermX* or MSYS245 or NEUWAVE* or Solero* or TATO or TATOPro*).tw,kf. (463)

38 (Evident* and Covidien).tw,kf. (6)

39 37 or 38 [MICROWAVE ABLATION DEVICES/SYSTEMS] (469)

40 19 or 36 or 39 [MICROWAVE ABLATION/COAGULATION] (10595)

41 6 and 40 [MICROWAVE ABLATION IN LIVER CANCER] (1171)

42 exp Animals/ not (exp Animals/ and Humans/) (4433505)

43 41 not 42 [ANIMAL-ONLY REMOVED] (1103)

44 (comment or editorial or interview or news or newspaper article).pt. (1243464)

45 (letter not (letter and randomized controlled trial)).pt. (975062)

46 43 not (44 or 45) [OPINION PIECES REMOVED] (1073)

47 limit 46 to systematic reviews (45)

48 meta analysis.pt. (85795)

49 exp meta-analysis as topic/ (16339)

50 (meta-analy* or metanaly* or metaanaly* or met analy* or integrative research or integrative review* or integrative overview* or research integration or research overview* or collaborative review*).tw,kf. (128107)

51 (systematic review* or systematic overview* or evidence-based review* or evidence-based overview* or (evidence adj3 (review* or overview*)) or meta-review* or meta-overview* or meta-synthes* or rapid review* or "review of reviews" or technology assessment* or HTA or HTAs).tw,kf. (158628)

52 exp Technology assessment, biomedical/ (10278)

53 (cochrane or health technology assessment or evidence report).jw. (15363)

54 ((indirect* or mixed or multi-treatment*) adj2 compar*).tw,kf. (4085)

55 ((network* or network-based) adj (MA or MAs)).tw,kf. (5)

56 or/48-55 (274350)

57 46 and 56 (31)

58 47 or 57 [SYSTEMATIC REVIEWS/META-ANALYSES] (51)

59 (controlled clinical trial or randomized controlled trial).pt. (542855)

60 "Clinical Trials as Topic".sh. (182891)

61 exp Randomized Controlled Trials as Topic/ (116639)

62 (randomi#ed or randomi#ation* or randomly or RCT$1 or placebo*).tw,kf. (840183)

63 ((singl* or doubl* or trebl* or tripl*) adj (mask* or blind* or dumm*)).tw,kf. (155371)

64 trial.ti. (179008)

65 or/59-64 (1237668)

66 46 and 65 [RCTS] (104)

67 58 or 66 [SRs/MAs, RCTs] (131)

68 (2017 10 25* or 2017 10 26* or 2017 10 27* or 2017 10 28* or 2017 10 29* or 2017 10 30* or 2017 10 31* or 2017 11* or 2017 12* or 2018*).dt. (481433)

**69 67 and 68 (5)**

***************************

Embase

Database: Embase <1988 to 2018 Week 11>

Search Strategy:

--------------------------------------------------------------------------------

1 exp liver cancer/ (186912)

2 (hepatoma* or hepatoblastoma* or hepato-blastoma*).tw,kw. (27153)

3 ((neoplas* or cancer* or tumour* or tumor* or carcinoma* or malignan* or metasta* or oncolog*) adj3 (liver* or hepat*)).tw,kw. (193052)

4 ((adenoma? or adenocarcinoma? or adeno-carcinoma? or blastoma? or carcinosarcoma? or carcino-sarcoma? or leukemia? or leukaemia? or lymphoma? or melanoma? or mesenchymoma? or mesothelioma? or sarcoma? or thymoma?) adj3 (liver* or hepat*)).tw,kw. (8967)

5 HCC.tw,kw. (68418)

6 or/1-5 [LIVER CANCER] (262834)

7 exp microwave thermotherapy/ (1230)

8 (microwave* adj3 (therap* or treatment*)).tw,kw. (1974)

9 MW ablation*.tw,kw. (187)

10 MW coagulation*.tw,kw. (2)

11 (MW adj (electrocoagulation* or electro-coagulation*)).tw,kw. (0)

12 (MW adj (thermocoagulation* or thermo-coagulation*)).tw,kw. (0)

13 (MW adj (electrocauter* or electro-cauter*)).tw,kw. (0)

14 (MW adj (thermal therap* or thermotherap* or thermo-therap*)).tw,kw. (3)

15 MWA.tw,kw. (1051)

16 (PMCT or PMCTs).tw,kw. (477)

17 (PMAT or PMATs).tw,kw. (154)

18 (RALTCT or RALTCTs).tw,kw. (1)

19 (MCT or MCTs).tw,kw. (7883)

20 or/7-19 (12208)

21 microwave radiation/ (17224)

22 microwave*.tw,kw. (30724)

23 (extremely high frequency adj (radiowave* or radio wave* or wave*)).tw,kw. (0)

24 (EHF adj (radiowave* or radio wave* or wave*)).tw,kw. (5)

25 ((ultrahigh or ultra-high) adj frequency adj (radiowave* or radio wave* or wave*)).tw,kw. (8)

26 or/21-25 [MICROWAVES] (33151)

27 ablation device/ (361)

28 ablation therapy/ (13896)

29 thermal ablation delivery device/ (47)

30 ablat*.tw,kw. (134269)

31 thermal heat*.tw,kw. (435)

32 (thermal therap* or thermotherap* or thermo-therap*).tw,kw. (4328)

33 electrocoagulation/ (5134)

34 coagulation*.tw,kw. (101044)

35 (electrocoagulation* or electro-coagulation*).tw,kw. (2696)

36 (thermocoagulation* or thermo-coagulation*).tw,kw. (1011)

37 (electrocauter* or electro-cauter*).tw,kw. (4460)

38 or/27-37 [ABLATION/COAGULATION] (246780)

39 26 and 38 (4251)

40 (Acculis* or Amica* or AMICA-GEM* or Avecure* or Certus* or Emblation* or Emprint* or FORSEA* or MicrothermX* or MSYS245 or NEUWAVE* or Solero* or TATO or TATOPro*).tw,kf,dv. (1128)

41 Evident.dv. (23)

42 (Evident* and Covidien).tw,kf. (33)

43 or/40-42 [MICROWAVE ABLATION DEVICES/SYSTEMS] (1177)

44 20 or 39 or 43 [MICROWAVE ABLATION/COAGULATION] (15341)

45 6 and 44 [MICROWAVE ABLATION IN LIVER CANCER] (1899)

46 exp animal experimentation/ or exp animal model/ or exp animal experiment/ or nonhuman/ or exp vertebrate/ (20577219)

47 exp human/ or exp human experimentation/ or exp human experiment/ (16270364)

48 46 not 47 (4307398)

49 45 not 48 [ANIMAL-ONLY REMOVED] (1806)

50 editorial.pt. (527702)

51 letter.pt. not (letter.pt. and randomized controlled trial/) (872634)

52 49 not (50 or 51) [OPINION PIECES REMOVED] (1763)

53 meta-analysis/ (139790)

54 "systematic review"/ (160784)

55 (meta-analy* or metanaly* or metaanaly* or met analy* or integrative research or integrative review* or integrative overview* or research integration or research overview* or collaborative review*).tw,kw. (167387)

56 (systematic review* or systematic overview* or evidence-based review* or evidence-based overview* or (evidence adj3 (review* or overview*)) or meta-review* or meta-overview* or meta-synthes* or rapid review* or "review of reviews" or technology assessment* or HTA or HTAs).tw,kw. (190513)

57 biomedical technology assessment/ (11996)

58 (cochrane or health technology assessment or evidence report).jw. (23221)

59 ((indirect* or mixed or multi-treatment*) adj2 compar*).tw,kw. (6115)

60 ((network* or network-based) adj (MA or MAs)).tw,kw. (12)

61 or/53-60 (383492)

62 52 and 61 [SYSTEMATIC REVIEWS/META-ANALYSES] (68)

63 randomized controlled trial/ or controlled clinical trial/ (650484)

64 exp "clinical trial (topic)"/ (259591)

65 (randomi#ed or randomi#ation* or randomly or RCT$1 or placebo*).tw,kw. (1107235)

66 ((singl* or doubl* or trebl* or tripl*) adj (mask* or blind* or dumm*)).tw,kw. (184887)

67 trial.ti. (222579)

68 or/63-67 (1576327)

69 52 and 68 [RCTs] (224)

70 62 or 69 [REVIEWS, RCTs] (248)

71 ("20171023" or "20171024" or "20171025" or "20171026" or "20171027" or "20171028" or "20171029" or "20171030" or "20171031" or 201711* or 201712* or 2018*).dc. (685695)

**72 70 and 71 (13)**

***************************

CENTRAL

Database: EBM Reviews - Cochrane Central Register of Controlled Trials <February 2018>, EBM Reviews - Cochrane Database of Systematic Reviews <2005 to March 14, 2018>

Search Strategy:

--------------------------------------------------------------------------------

1 exp Liver Neoplasms/ (1891)

2 (hepatoma* or hepatoblastoma* or hepato-blastoma*).ti,ab,kw. (157)

3 ((neoplas* or cancer* or tumour* or tumor* or carcinoma* or malignan* or metasta* or oncolog*) adj3 (liver* or hepat*)).ti,ab,kw. (5501)

4 ((adenoma? or adenocarcinoma? or adeno-carcinoma? or blastoma? or carcinosarcoma? or carcino-sarcoma? or leukemia? or leukaemia? or lymphoma? or melanoma? or mesenchymoma? or mesothelioma? or sarcoma? or thymoma?) adj3 (liver* or hepat*)).ti,ab,kw. (200)

5 HCC.ti,ab,kw. (1806)

6 or/1-5 [LIVER CANCER] (6196)

7 (microwave* adj3 (therap* or treatment*)).ti,ab,kw. (189)

8 MW ablation*.ti,ab,kw. (11)

9 MW coagulation*.ti,ab,kw. (0)

10 (MW adj (electrocoagulation* or electro-coagulation*)).ti,ab,kw. (0)

11 (MW adj (thermocoagulation* or thermo-coagulation*)).ti,ab,kw. (0)

12 (MW adj (electrocauter* or electro-cauter*)).ti,ab,kw. (0)

13 (MW adj (thermal therap* or thermotherap* or thermo-therap*)).ti,ab,kw. (0)

14 MWA.ti,ab,kw. (52)

15 (PMCT or PMCTs).ti,ab,kw. (12)

16 (PMAT or PMATs).ti,ab,kw. (2)

17 (RALTCT or RALTCTs).ti,ab,kw. (0)

18 (MCT or MCTs).ti,ab,kw. (666)

19 or/7-18 (911)

20 Microwaves/ (200)

21 microwave*.ti,ab,kw. (509)

22 (extremely high frequency adj (radiowave* or radio wave* or wave*)).ti,ab,kw. (0)

23 (EHF adj (radiowave* or radio wave* or wave*)).ti,ab,kw. (4)

24 ((ultrahigh or ultra-high) adj frequency adj (radiowave* or radio wave* or wave*)).ti,ab,kw. (0)

25 or/20-24 [MICROWAVES] (551)

26 exp Ablation Techniques/ (5055)

27 ablat*.ti,ab,kw. (5237)

28 thermal heat*.ti,ab,kw. (23)

29 (thermal therap* or thermotherap* or thermo-therap*).ti,ab,kw. (339)

30 Electrocoagulation/ (648)

31 coagulation*.ti,ab,kw. (5183)

32 (electrocoagulation* or electro-coagulation*).ti,ab,kw. (218)

33 (thermocoagulation* or thermo-coagulation*).ti,ab,kw. (101)

34 (electrocauter* or electro-cauter*).ti,ab,kw. (482)

35 or/26-34 [ABLATION/COAGULATION] (14154)

36 25 and 35 (242)

37 (Acculis* or Amica* or AMICA-GEM* or Avecure* or Certus* or Emblation* or Emprint* or FORSEA* or MicrothermX* or MSYS245 or NEUWAVE* or Solero* or TATO or TATOPro*).ti,ab,kw. (30)

38 (Evident* and Covidien).ti,ab,kw. (0)

39 37 or 38 [MICROWAVE ABLATION DEVICES/SYSTEMS] (30)

40 19 or 36 or 39 [MICROWAVE ABLATION/COAGULATION] (1062)

41 6 and 40 [MICROWAVE ABLATION IN LIVER CANCER] (74)

42 ("201711" or "201712" or 2018*).up. (267872)

43 41 and 42 (19)

**44 43 use cctr (19)**

***************************

*Non-RCTs*

MEDLINE

Database: Ovid MEDLINE(R) Epub Ahead of Print, In-Process & Other Non-Indexed Citations, Ovid MEDLINE(R) Daily and Ovid MEDLINE(R) <1946 to Present>

Search Strategy:

--------------------------------------------------------------------------------

1 exp neoplasms/ (3019512)

2 (hepatoma* or hepatoblastoma* or hepato-blastoma*).tw,kf. (30354)

3 (neoplas* or cancer* or tumour* or tumor* or carcinoma* or malignan* or metasta* or oncolog*).tw,kf. (2985926)

4 (adenoma? or adenocarcinoma? or adeno-carcinoma? or blastoma? or carcinosarcoma? or carcino-sarcoma? or leukemia? or leukaemia? or lymphoma? or melanoma? or mesenchymoma? or mesothelioma? or sarcoma? or thymoma?).tw,kf. (740037)

5 HCC.tw,kf. (43282)

6 or/1-5 [CANCER] (3938519)

7 (microwave* adj3 (therap* or treatment*)).tw,kf. (1656)

8 MW ablation*.tw,kf. (95)

9 MW coagulation*.tw,kf. (1)

10 (MW adj (electrocoagulation* or electro-coagulation*)).tw,kf. (0)

11 (MW adj (thermocoagulation* or thermo-coagulation*)).tw,kf. (0)

12 (MW adj (electrocauter* or electro-cauter*)).tw,kf. (0)

13 (MW adj (thermal therap* or thermotherap* or thermo-therap*)).tw,kf. (3)

14 MWA.tw,kw. (580)

15 (PMCT or PMCTs).tw,kf. (326)

16 (PMAT or PMATs).tw,kf. (89)

17 (RALTCT or RALTCTs).tw,kf. (1)

18 (MCT or MCTs).tw,kf. (5504)

19 or/7-18 (8053)

20 Microwaves/ (15399)

21 microwave*.tw,kf. (30647)

22 (extremely high frequency adj (radiowave* or radio wave* or wave*)).tw,kf. (0)

23 (EHF adj (radiowave* or radio wave* or wave*)).tw,kf. (6)

24 ((ultrahigh or ultra-high) adj frequency adj (radiowave* or radio wave* or wave*)).tw,kf. (15)

25 or/20-24 [MICROWAVES] (33236)

26 exp Ablation Techniques/ (103925)

27 ablat*.tw,kf. (94738)

28 thermal heat*.tw,kf. (394)

29 (thermal therap* or thermotherap* or thermo-therap*).tw,kf. (3410)

30 Electrocoagulation/ (11077)

31 coagulation*.tw,kf. (90180)

32 (electrocoagulation* or electro-coagulation*).tw,kf. (3438)

33 (thermocoagulation* or thermo-coagulation*).tw,kf. (936)

34 (electrocauter* or electro-cauter*).tw,kf. (3318)

35 or/26-34 [ABLATION/COAGULATION] (256508)

36 25 and 35 (3171)

37 (Acculis* or Amica* or AMICA-GEM* or Avecure* or Certus* or Emblation* or Emprint* or FORSEA* or MicrothermX* or MSYS245 or NEUWAVE* or Solero* or TATO or TATOPro*).tw,kf. (463)

38 (Evident* and Covidien).tw,kf. (6)

39 37 or 38 [MICROWAVE ABLATION DEVICES/SYSTEMS] (469)

40 19 or 36 or 39 [MICROWAVE ABLATION/COAGULATION] (10592)

41 6 and 40 [MICROWAVE ABLATION IN CANCER] (3539)

42 exp Animals/ not (exp Animals/ and Humans/) (4433505)

43 41 not 42 [ANIMAL-ONLY REMOVED] (3052)

44 (comment or editorial or interview or news or newspaper article).pt. (1243464)

45 (letter not (letter and randomized controlled trial)).pt. (975062)

46 43 not (44 or 45) [OPINION PIECES REMOVED] (2969)

47 controlled clinical trial.pt. (92230)

48 Controlled Clinical Trial/ or Controlled Clinical Trials as Topic/ (97493)

49 (control* adj2 trial*).tw,kf. (219072)

50 Non-Randomized Controlled Trials as Topic/ (297)

51 (nonrandom* or non-random* or quasi-random* or quasi-experiment*).tw,kf. (46831)

52 (nRCT or nRCTs or non-RCT?).tw,kf. (613)

53 Controlled Before-After Studies/ (307)

54 (control* adj3 ("before and after" or "before after")).tw,kf. (3699)

55 Interrupted Time Series Analysis/ (390)

56 time series.tw,kf. (24341)

57 (pre- adj3 post-).tw,kf. (65321)

58 (pretest adj3 posttest).tw,kf. (4421)

59 Historically Controlled Study/ (132)

60 (control* adj2 stud$3).tw,kf. (202165)

61 Control Groups/ (1576)

62 (control* adj2 group$1).tw,kf. (427711)

63 trial.ti. (179008)

64 or/47-63 (1059967)

65 46 and 64 [NON-RCTS] (170)

66 exp Cohort Studies/ (1718747)

67 cohort?.tw,kf. (448777)

68 Retrospective Studies/ (675095)

69 (longitudinal or prospective or retrospective).tw,kf. (1043153)

70 ((followup or follow-up) adj (study or studies)).tw,kf. (46511)

71 Observational study.pt. (44780)

72 (observation$2 adj (study or studies)).tw,kf. (80687)

73 ((population or population-based) adj (study or studies or analys#s)).tw,kf. (15074)

74 ((multidimensional or multi-dimensional) adj (study or studies)).tw,kf. (100)

75 Comparative Study.pt. (1792103)

76 ((comparative or comparison) adj (study or studies)).tw,kf. (102643)

77 exp Case-Control Studies/ (901318)

78 ((case-control* or case-based or case-comparison) adj (study or studies)).tw,kf. (91016)

79 or/66-78 (3989935)

80 46 and 79 [OBSERVATIONAL STUDIES] (805)

81 65 or 80 [NON-RCTS, OBSERV STUDIES] (901)

82 (2017 11 2* or 2017 11 30 or 2017 12* or 2018*).dt. (385957)

**83 81 and 82 (29)**

***************************

Embase

Database: Embase <1988 to 2018 Week 11>

Search Strategy:

--------------------------------------------------------------------------------

1 exp liver cancer/ (186912)

2 (hepatoma* or hepatoblastoma* or hepato-blastoma*).tw,kw. (27153)

3 ((neoplas* or cancer* or tumour* or tumor* or carcinoma* or malignan* or metasta* or oncolog*) adj3 (liver* or hepat*)).tw,kw. (193052)

4 ((adenoma? or adenocarcinoma? or adeno-carcinoma? or blastoma? or carcinosarcoma? or carcino-sarcoma? or leukemia? or leukaemia? or lymphoma? or melanoma? or mesenchymoma? or mesothelioma? or sarcoma? or thymoma?) adj3 (liver* or hepat*)).tw,kw. (8967)

5 HCC.tw,kw. (68418)

6 or/1-5 [LIVER CANCER] (262834)

7 exp microwave thermotherapy/ (1230)

8 (microwave* adj3 (therap* or treatment*)).tw,kw. (1974)

9 MW ablation*.tw,kw. (187)

10 MW coagulation*.tw,kw. (2)

11 (MW adj (electrocoagulation* or electro-coagulation*)).tw,kw. (0)

12 (MW adj (thermocoagulation* or thermo-coagulation*)).tw,kw. (0)

13 (MW adj (electrocauter* or electro-cauter*)).tw,kw. (0)

14 (MW adj (thermal therap* or thermotherap* or thermo-therap*)).tw,kw. (3)

15 MWA.tw,kw. (1051)

16 (PMCT or PMCTs).tw,kw. (477)

17 (PMAT or PMATs).tw,kw. (154)

18 (RALTCT or RALTCTs).tw,kw. (1)

19 (MCT or MCTs).tw,kw. (7883)

20 or/7-19 (12208)

21 microwave radiation/ (17224)

22 microwave*.tw,kw. (30724)

23 (extremely high frequency adj (radiowave* or radio wave* or wave*)).tw,kw. (0)

24 (EHF adj (radiowave* or radio wave* or wave*)).tw,kw. (5)

25 ((ultrahigh or ultra-high) adj frequency adj (radiowave* or radio wave* or wave*)).tw,kw. (8)

26 or/21-25 [MICROWAVES] (33151)

27 ablation device/ (361)

28 ablation therapy/ (13896)

29 thermal ablation delivery device/ (47)

30 ablat*.tw,kw. (134269)

31 thermal heat*.tw,kw. (435)

32 (thermal therap* or thermotherap* or thermo-therap*).tw,kw. (4328)

33 electrocoagulation/ (5134)

34 coagulation*.tw,kw. (101044)

35 (electrocoagulation* or electro-coagulation*).tw,kw. (2696)

36 (thermocoagulation* or thermo-coagulation*).tw,kw. (1011)

37 (electrocauter* or electro-cauter*).tw,kw. (4460)

38 or/27-37 [ABLATION/COAGULATION] (246780)

39 26 and 38 (4251)

40 (Acculis* or Amica* or AMICA-GEM* or Avecure* or Certus* or Emblation* or Emprint* or FORSEA* or MicrothermX* or MSYS245 or NEUWAVE* or Solero* or TATO or TATOPro*).tw,kf,dv. (1128)

41 Evident.dv. (23)

42 (Evident* and Covidien).tw,kf. (33)

43 or/40-42 [MICROWAVE ABLATION DEVICES/SYSTEMS] (1177)

44 20 or 39 or 43 [MICROWAVE ABLATION/COAGULATION] (15341)

45 6 and 44 [MICROWAVE ABLATION IN LIVER CANCER] (1899)

46 exp animal experimentation/ or exp animal model/ or exp animal experiment/ or nonhuman/ or exp vertebrate/ (20577219)

47 exp human/ or exp human experimentation/ or exp human experiment/ (16270364)

48 46 not 47 (4307398)

49 45 not 48 [ANIMAL-ONLY REMOVED] (1806)

50 editorial.pt. (527702)

51 letter.pt. not (letter.pt. and randomized controlled trial/) (872634)

52 49 not (50 or 51) [OPINION PIECES REMOVED] (1763)

53 exp controlled clinical trial/ (650484)

54 exp "controlled clinical trial (topic)"/ (146341)

55 (control* adj2 trial*).tw,kw. (283497)

56 (nonrandom* or non-random* or quasi-random* or quasi-experiment*).tw,kw. (55509)

57 (nRCT or nRCTs or non-RCT$1).tw,kw. (905)

58 (control* adj3 ("before and after" or "before after")).tw,kw. (4452)

59 time series analysis/ (20308)

60 time series.tw,kw. (26680)

61 pretest posttest control group design/ (331)

62 (pre- adj3 post-).tw,kw. (109497)

63 (pretest adj3 posttest).tw,kw. (4669)

64 controlled study/ (5744065)

65 (control* adj2 stud$3).tw,kw. (252068)

66 control group/ (110986)

67 (control* adj2 group$1).tw,kw. (567250)

68 trial.ti. (222579)

69 or/53-68 (6488130)

70 52 and 69 [NON-RCTS] (466)

71 cohort analysis/ (348331)

72 cohort?.tw,kw. (730573)

73 retrospective study/ (607404)

74 longitudinal study/ (107116)

75 prospective study/ (422618)

76 (longitudinal or prospective or retrospective).tw,kw. (1487347)

77 follow up/ (1216139)

78 ((followup or follow-up) adj (study or studies)).tw,kw. (51871)

79 observational study/ (132688)

80 (observation$2 adj (study or studies)).tw,kw. (123453)

81 population research/ (88049)

82 ((population or population-based) adj (study or studies or analys#s)).tw,kw. (19673)

83 ((multidimensional or multi-dimensional) adj (study or studies)).tw,kw. (106)

84 exp comparative study/ (986739)

85 ((comparative or comparison) adj (study or studies)).tw,kw. (95011)

86 exp case control study/ (139912)

87 ((case-control* or case-based or case-comparison) adj (study or studies)).tw,kw. (113074)

88 or/71-87 (4116402)

89 52 and 88 [OBSERVATIONAL STUDIES] (806)

90 70 or 89 [NON-RCTS, OBSERVATIONAL STUDIES] (1006)

91 (2017112* or "20171130" or 201712* or 2018*).dc. (546328)

**92 90 and 91 (44)**
