# Supplementary material for: Microwave ablation compared with hepatic resection for the treatment of hepatocellular carcinoma and liver metastases: a systematic review and meta-analysis
Source: World J Surg Oncol. 2019 Jun 10;17:98. doi: 10.1186/s12957-019-1632-6 (PMC6558848; doi:10.1186/s12957-019-1632-6)
Supplement: Supplementary file 3 — Study treatment selection criteria and resectability. Table describing the selection criteria used to assign patients to different treatments and descriptions of whether MWA patients were resectable. (DOCX 31 kb) [file 12957_2019_1632_MOESM3_ESM.docx]

| Study | Comparators | Resectability of patients | Selection criteria for assigning patients to a specific treatment |
| --- | --- | --- | --- |
| Xu 2015 | HR  MWA | - | - |
| Tanaka 2006 | HR | “To overcome subjectivity in definition of resectibility, patients with 5 or more liver nodules in a bilobar distribution were included and determined to be eligible for the study” | Low prediction scores (variables: resection fraction, age, indocyanine green retention rate) |
|  | HR+ MWA |  | Patients unresectable even using portal vein embolization or 2-stage hepatectomy |
| Wang 2008 | HR  MWA | - | - |
| Imura 2012 | HR  MWA + HR | - | - |
| Stattner 2013 | MWA  MWA + HR | “Prior to surgery, all patients were discussed at a supraregional multidisciplinary tumour board (sMDT) and considered technically unresectable at a single procedure.” |  |
| Takami 2013 | HR |  | HCC >3cm, tumors at surface |
|  | MWA |  | HCC ≤3cm, <10 tumors, poor liver function/performance status |
| Shi 2014 | MWA  HR | - | - |
| Tan 2014 | HR  MWA + HR | - | - |
| Zhang 2016 | HR |  | Superficial tumors, MWA cannot be safely or effectively performed |
|  | MWA |  | Tumors deep in the liver, sever cirrhosis or portal hypertension, comorbidities preventing patient from tolerating operation |
| Li 2017 | HR |  | Tumor at the edge of the liver or deep in the liver and close to the major hepatic vasculature |
|  | MWA |  | Tumor deep in the liver, but far from hepatic vasculature |
| Philips 2017 | MWA  HR | “Tumors were regarded as resectable if the anticipated hepatic parenchymal transection plane yielded a tumour-free margin while preserving adequate hepatic remnant.” |  |
| Ryu 2017 | HR | “As some patients tended to prefer the less invasive treatment to the more aggressive resection, they were treated by MCN even when the hepatic lesion was resectable” | Sufficient hepatic reserve |
|  | MWA |  | Metastases <3cm, ineligible for resection due to comorbidities, preference for less invasive treatment |
| Song 2017 | HR  MWA | “Traditionally, CRLM with the size more than 5cm cannot be treated using liver resection, thus CRLM with the size more than 5cm is defined as unresectable CRLM.” |  |
| Zhang 2017 | HR |  | Patient preference |
|  | MWA |  | Centrally located tumors, patient preference |
| Chen 2018 | HR |  | Patient preference |
|  | MWA + HR |  | Patient preference |
| Chong 2018 | HR |  | Subcapsular, solitary or oligonodular tumor, sufficient liver remnant |
|  | MWA |  | Small and/or deep-seated intra-parenchymal tumor |

Abbreviations: CRLM, colorectal liver metastases; HCC, hepatocellular carcinoma; HR, hepatic resection; MCN, microwave coagulo-necrotic therapy; MWA, microwave ablation
