# Supplementary figures and images for: Microwave ablation compared with hepatic resection for the treatment of hepatocellular carcinoma and liver metastases: a systematic review and meta-analysis
Source: World J Surg Oncol. 2019 Jun 10;17:98. doi: 10.1186/s12957-019-1632-6 (PMC6558848; doi:10.1186/s12957-019-1632-6)

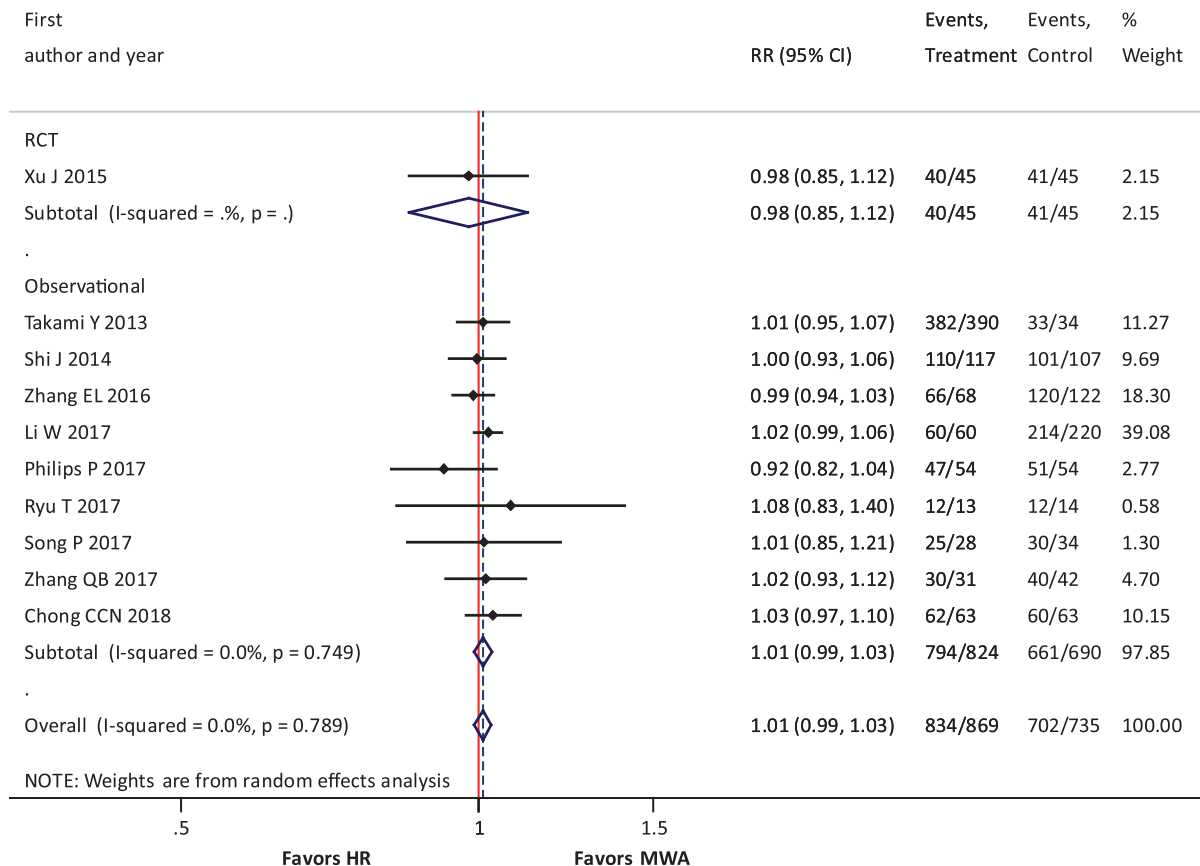

Supplement: Supplementary file 5 — Forest plot of random effects meta-analysis results for 1-year OS (P = 0.409). Forest plot of secondary outcome data. Abbreviations: OS, overall survival (PDF 3082 kb) [file 12957_2019_1632_MOESM5_ESM.pdf]

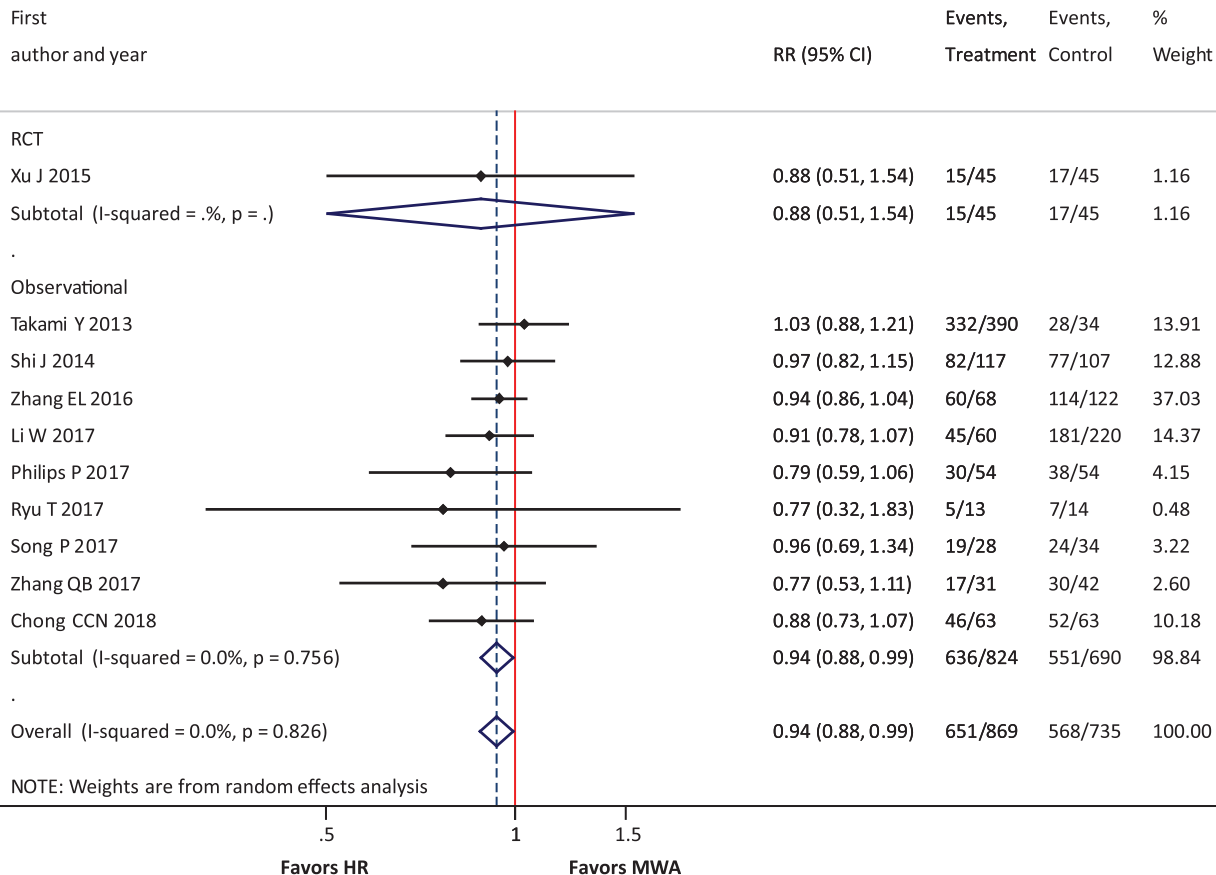

Supplement: Supplementary file 6 — Forest plot of random effects meta-analysis results for 3-year OS (P = 0.03). Forest plot of secondary outcome data. Abbreviations: OS, overall survival (PDF 3075 kb) [file 12957_2019_1632_MOESM6_ESM.pdf]

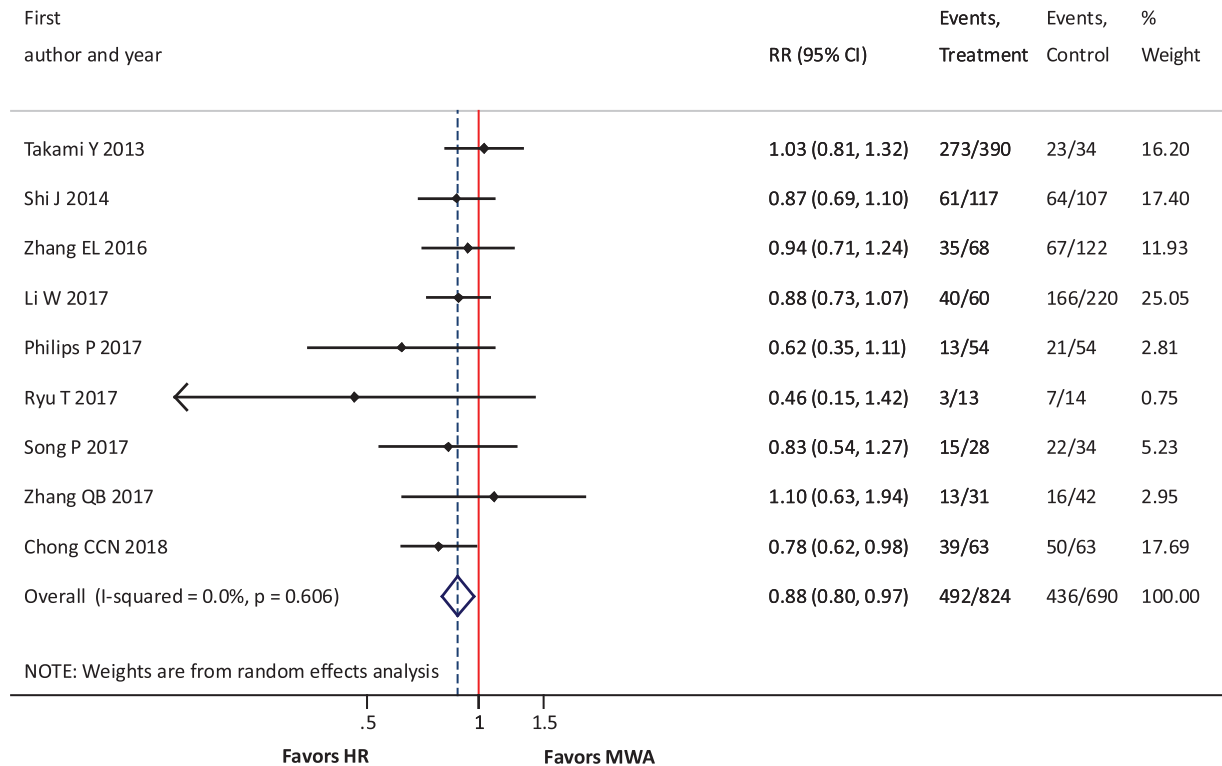

Supplement: Supplementary file 7 — Forest plot of random effects meta-analysis results for 5-year OS (P = 0.01). Forest plot of secondary outcome data. Abbreviations: OS, overall survival (PDF 2537 kb) [file 12957_2019_1632_MOESM7_ESM.pdf]

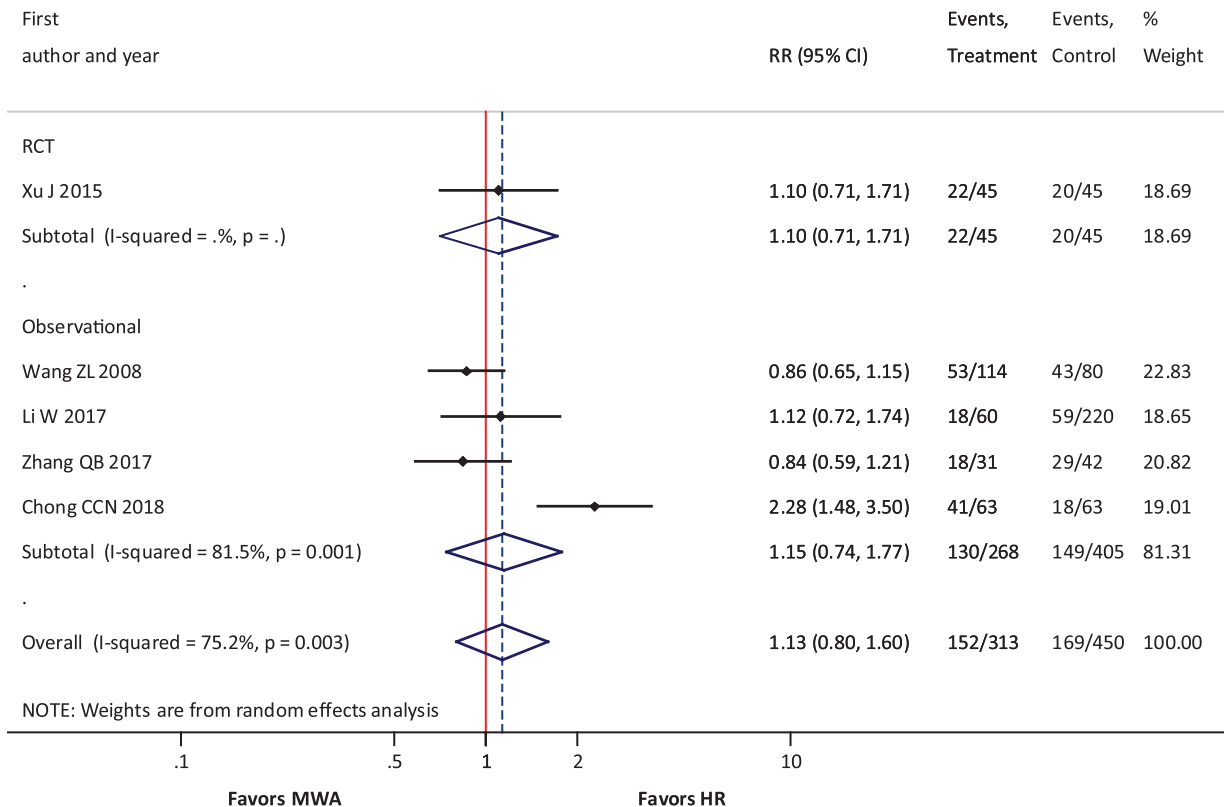

Supplement: Supplementary file 8 — Forest plot of random effects meta-analysis results for IDL (P = 0.474). Forest plot of secondary outcome data. Abbreviations: IDL, intrahepatic de novo lesions (PDF 2383 kb) [file 12957_2019_1632_MOESM8_ESM.pdf]

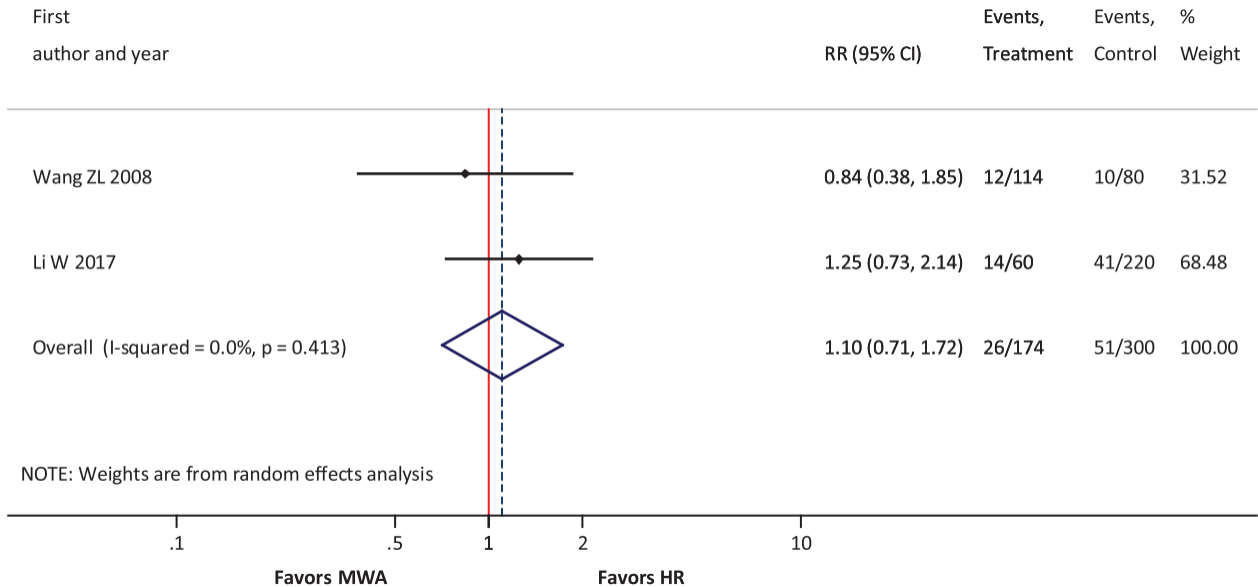

Supplement: Supplementary file 9 — Forest plot of random effects meta-analysis results for EHM (P = 0.659). Forest plot of secondary outcome data. Abbreviations: EHM, extrahepatic metastasis (PDF 1517 kb) [file 12957_2019_1632_MOESM9_ESM.pdf]

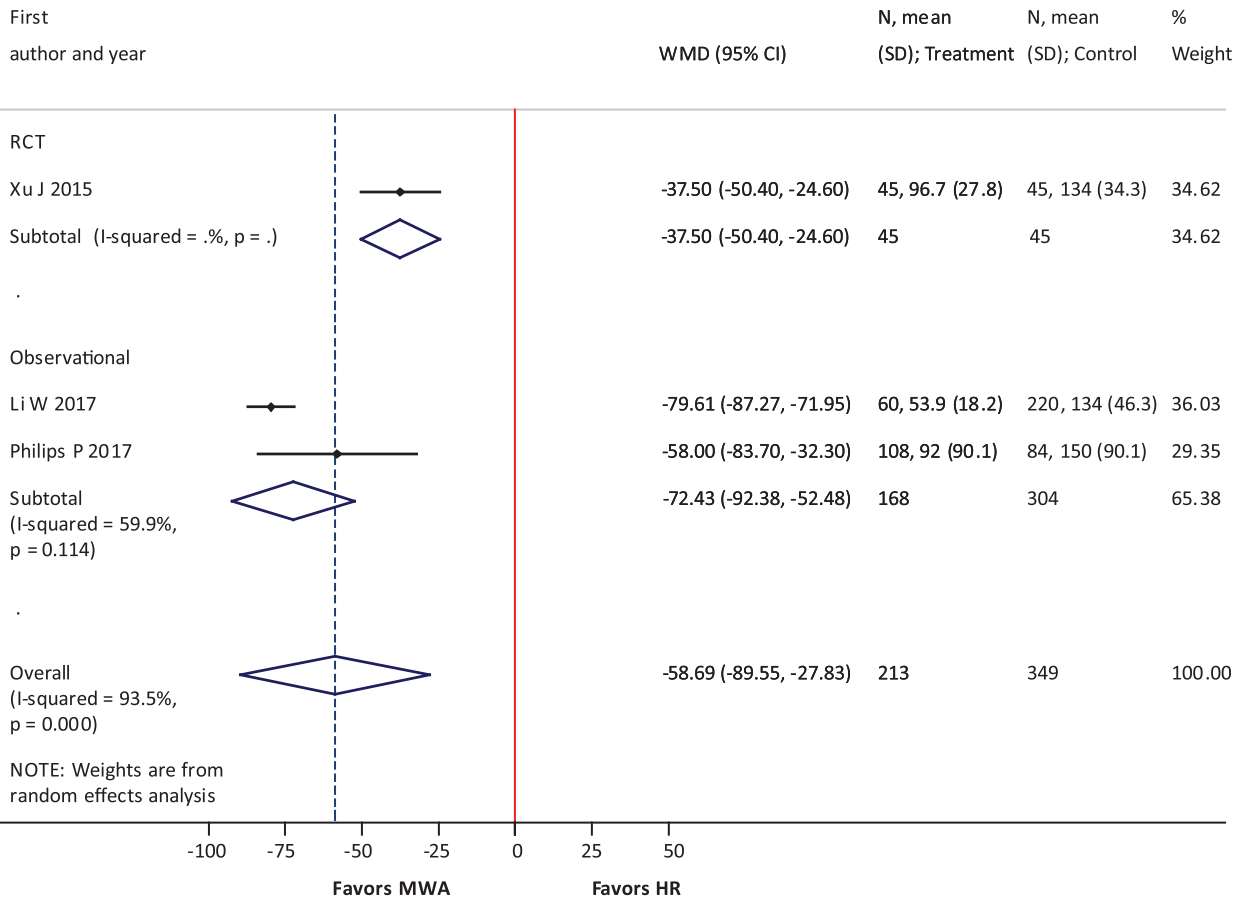

Supplement: Supplementary file 10 — Forest plot of random effects meta-analysis results for operative time (P < 0.001). Forest plot of secondary outcome data. (PDF 2454 kb) [file 12957_2019_1632_MOESM10_ESM.pdf]

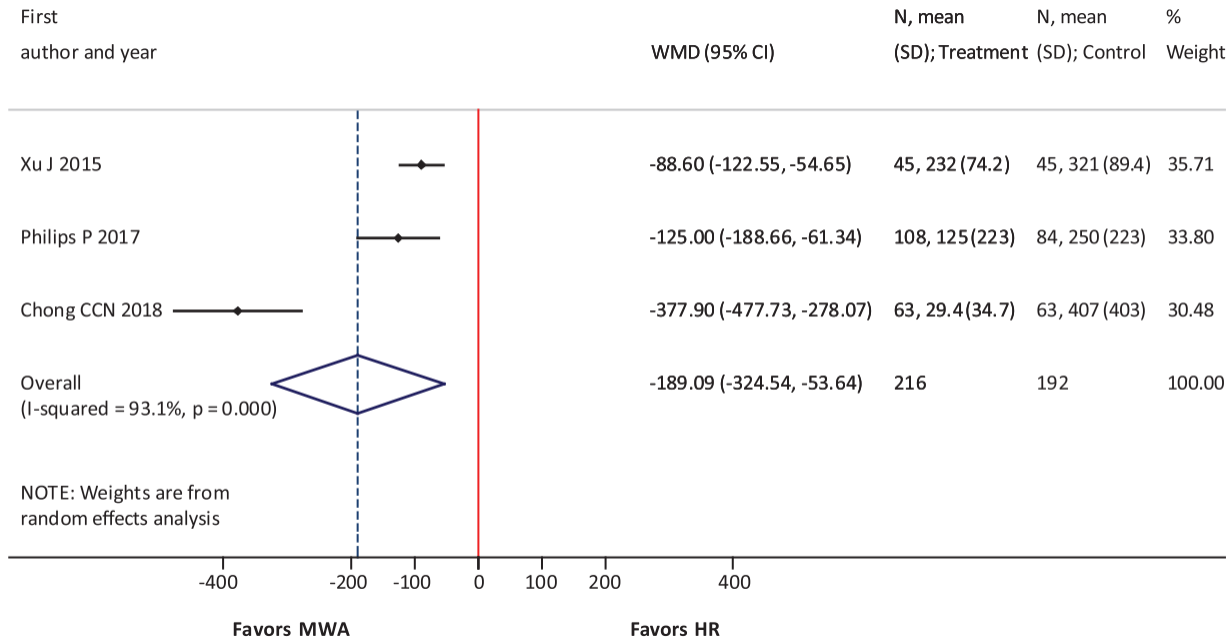

Supplement: Supplementary file 11 — Forest plot of random effects meta-analysis results for intraoperative blood loss (P = 0.006). Forest plot of secondary outcome data. (PDF 2046 kb) [file 12957_2019_1632_MOESM11_ESM.pdf]

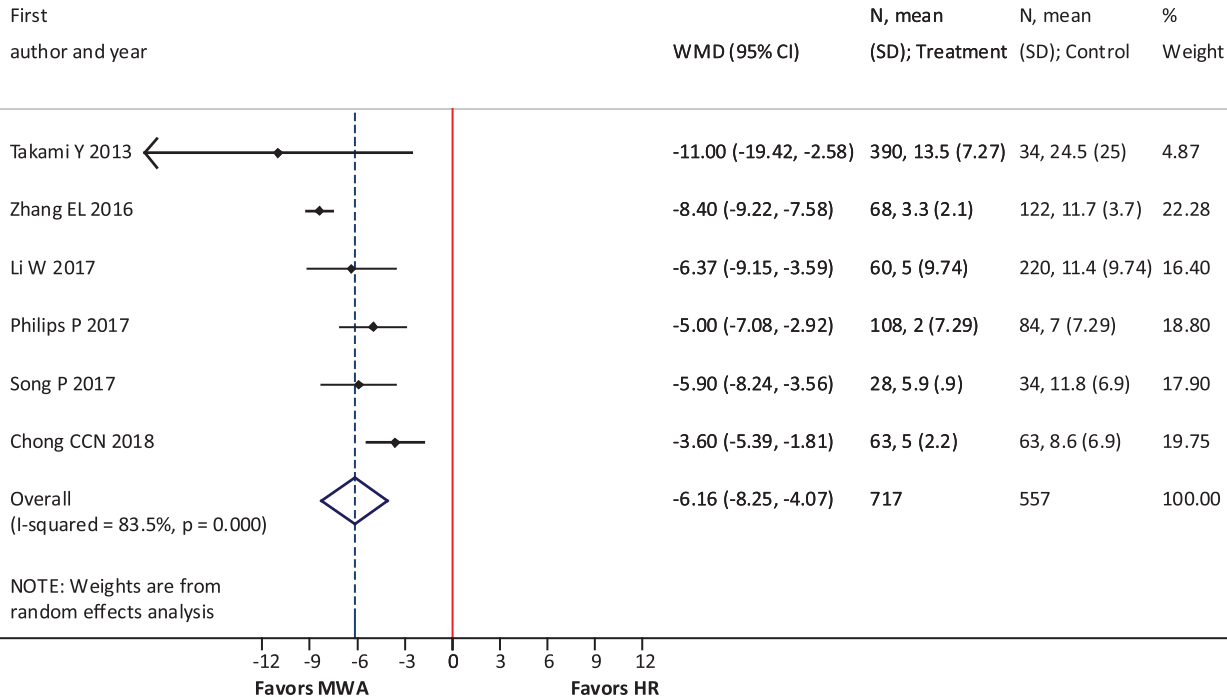

Supplement: Supplementary file 12 — Forest plot of random effects meta-analysis results for LOS (P < 0.001). Forest plot of secondary outcome data. Abbreviations: LOS, length of stay (PDF 2418 kb) [file 12957_2019_1632_MOESM12_ESM.pdf]

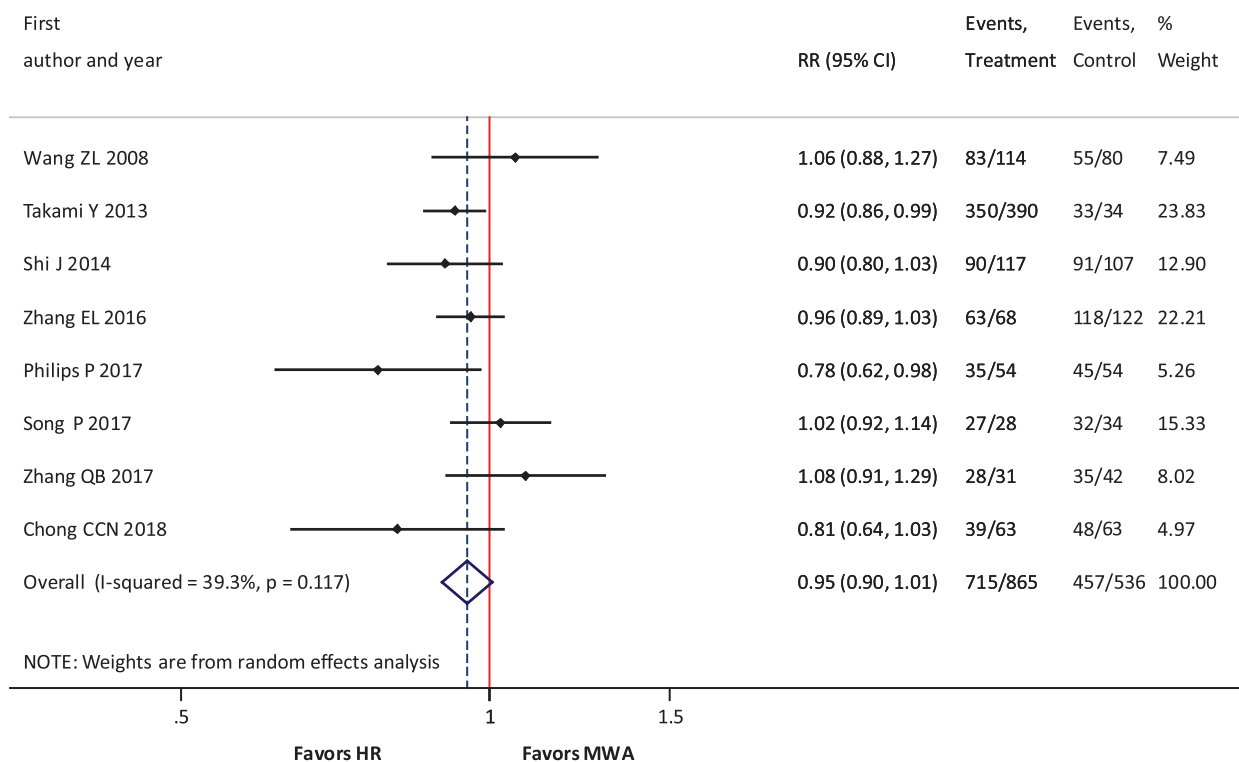

Supplement: Supplementary file 13 — Forest plot of random effects meta-analysis results for 1-year DFS (P = 0.085). Forest plot of secondary outcome data. Abbreviations: DFS, disease-free survival (PDF 2422 kb) [file 12957_2019_1632_MOESM13_ESM.pdf]

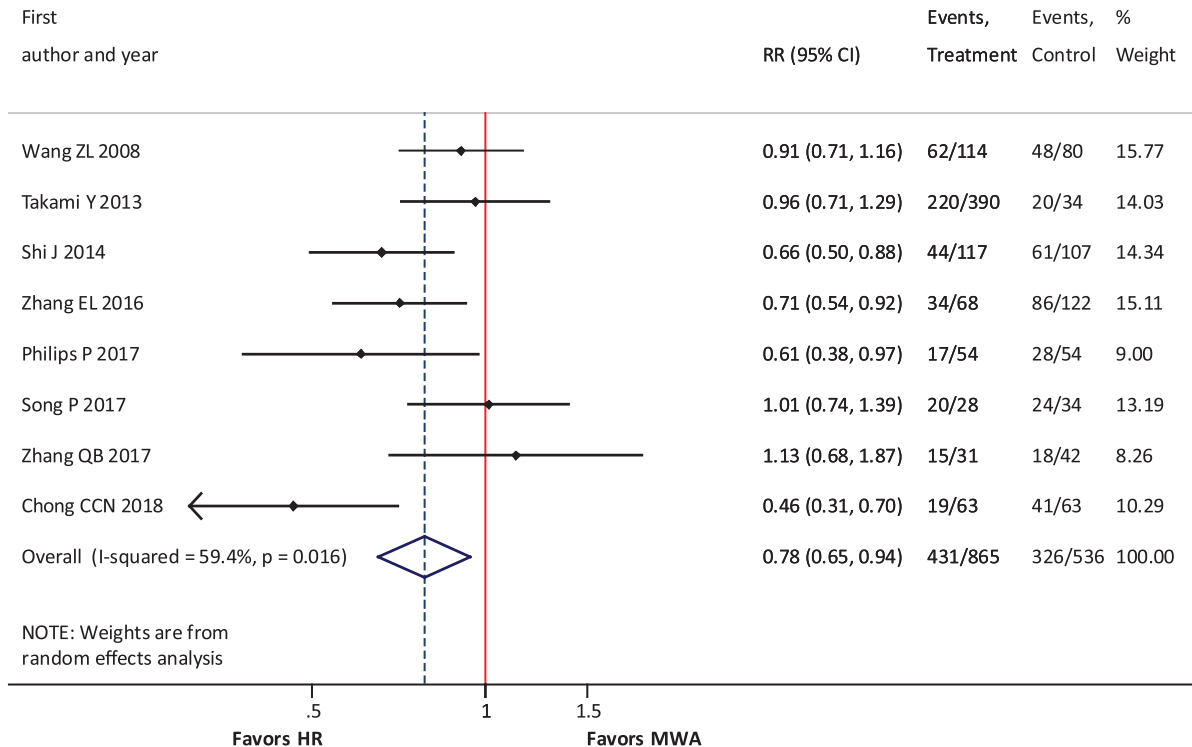

Supplement: Supplementary file 14 — Forest plot of random effects meta-analysis results for 3-year DFS (P = 0.009). Forest plot of secondary outcome data. Abbreviations: DFS, disease-free survival (PDF 2412 kb) [file 12957_2019_1632_MOESM14_ESM.pdf]

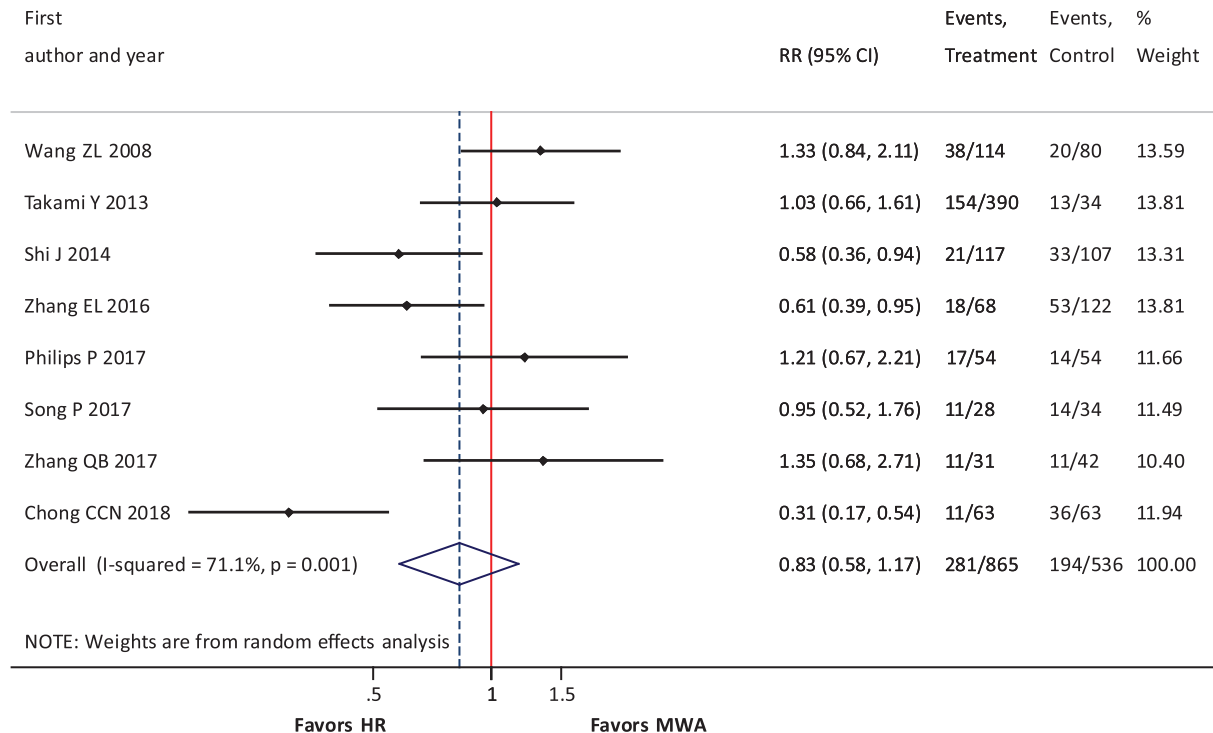

Supplement: Supplementary file 15 — Forest plot of random effects meta-analysis results for 5-year DFS (P = 0.284). Forest plot of secondary outcome data. Abbreviations: DFS, disease-free survival (PDF 2391 kb) [file 12957_2019_1632_MOESM15_ESM.pdf]

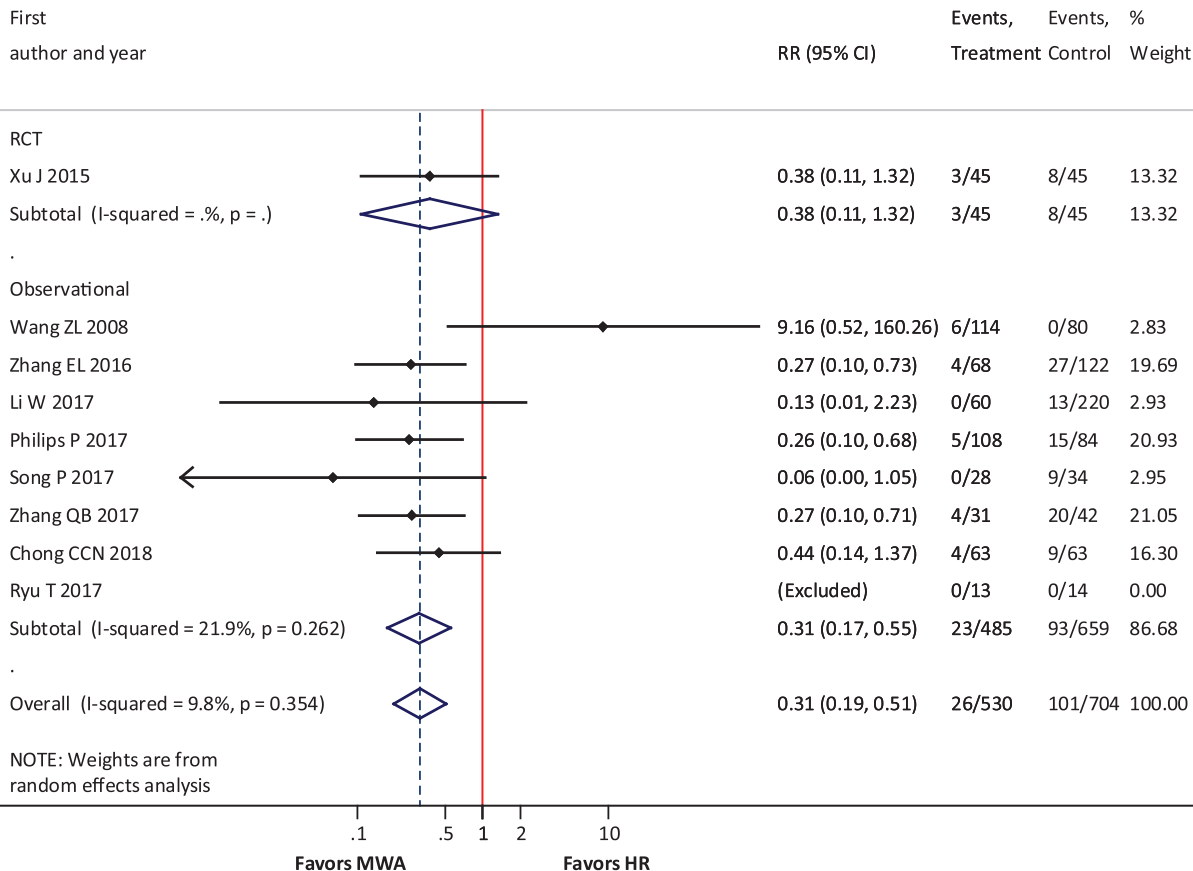

Supplement: Supplementary file 16 — Forest plot of random effects meta-analysis results for overall complications (P < 0.001). Forest plot of secondary outcome data. (PDF 2903 kb) [file 12957_2019_1632_MOESM16_ESM.pdf]

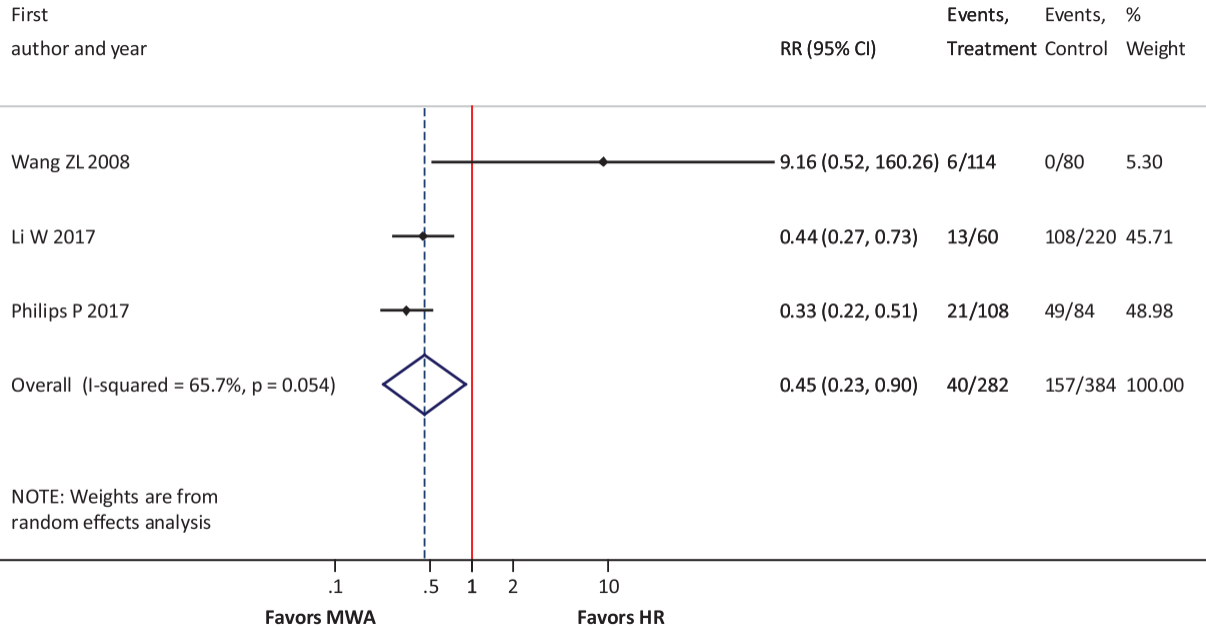

Supplement: Supplementary file 18 — Forest plot of random effects meta-analysis results for minor complications (P = 0.024). Forest plot of secondary outcome data. (PDF 1674 kb) [file 12957_2019_1632_MOESM18_ESM.pdf]

(a)

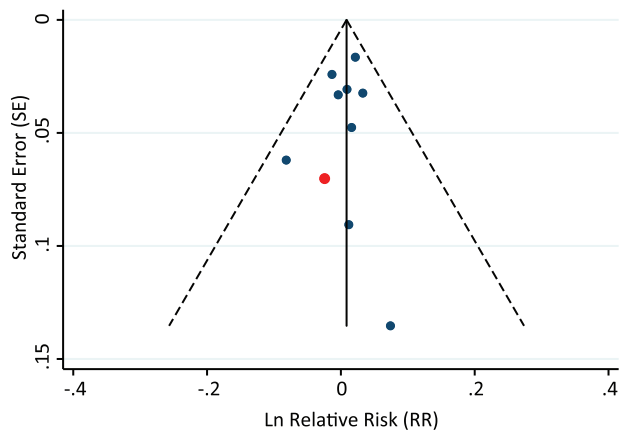

(b)

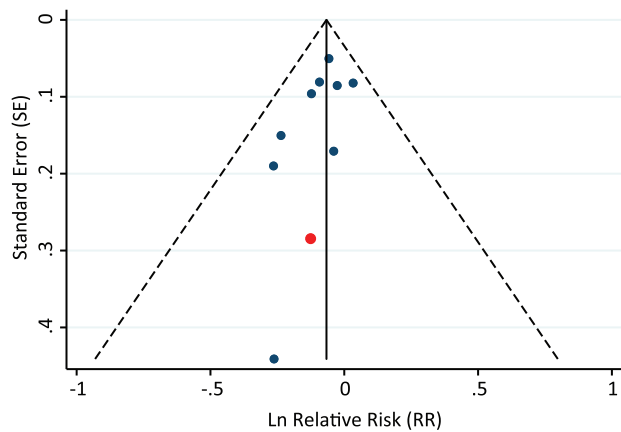

(c)

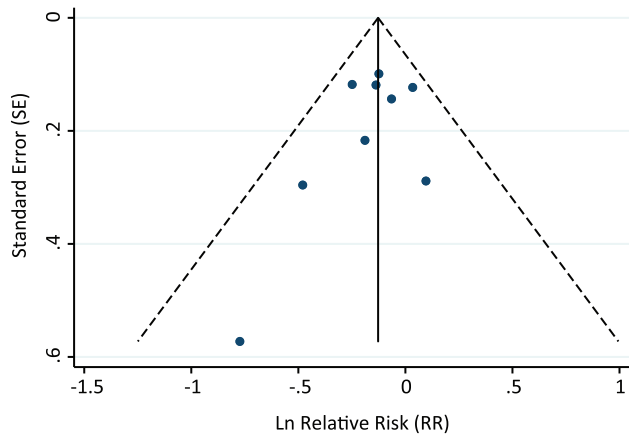

(d)

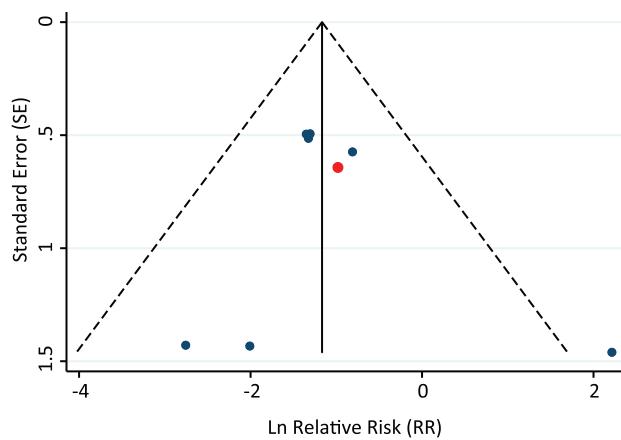

Supplement: Supplementary file 19 — Funnel plots assessing publication bias. Funnel plots for secondary outcome publication bias assessment. (a) 1-year OS (n = 10), (b) 3-year OS (n = 10), (c) 5-year OS (n = 9), and (d) overall complications (n = 8). Red dots indicate the RCT. Abbreviations: OS, overall survival; RCT, randomized controlled trial. (PDF 1214 kb) [file 12957_2019_1632_MOESM19_ESM.pdf]
