# Supplementary material for: Microwave ablation compared with hepatic resection for the treatment of hepatocellular carcinoma and liver metastases: a systematic review and meta-analysis
Source: World J Surg Oncol. 2019 Jun 10;17:98. doi: 10.1186/s12957-019-1632-6 (PMC6558848; doi:10.1186/s12957-019-1632-6)
Supplement: Supplementary file 17 — Forest plot of random effects meta-analysis results for major complications (P = 0.002). Forest plot of secondary outcome data. (PDF 1739 kb) [file 12957_2019_1632_MOESM17_ESM.pdf]

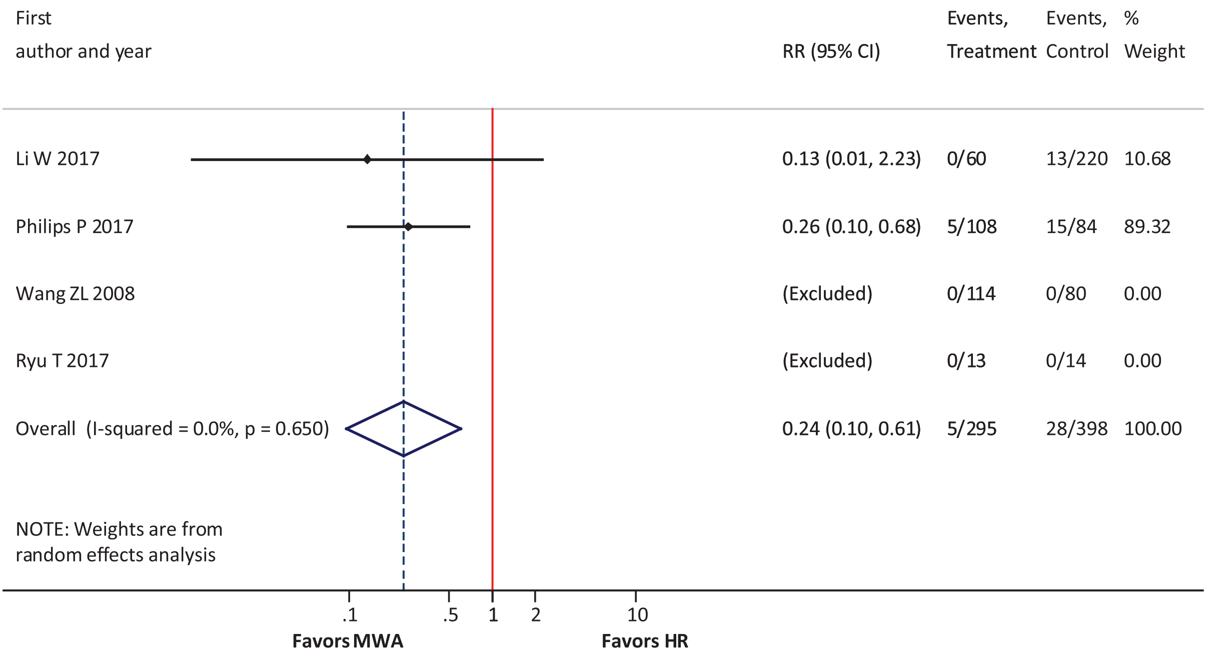
First
author and year
RR (95% CI)
Events,
Treatment
Events,
Control
%
Weight
Li W 2017
0.13 (0.01, 2.23)
0/60
13/220
10.68
Philips P 2017
0.26 (0.10, 0.68)
5/108
15/84
89.32
Wang ZL 2008
(Excluded)
0/114
0/80
0.00
Ryu T 2017
(Excluded)
0/13
0/14
0.00
Overall (I-squared = 0.0%, p = 0.650)
0.24 (0.10, 0.61)
5/295
28/398
100.00
NOTE: Weights are from
random effects analysis
.1
.5
1
2
10
Favors MWA
Favors HR
